# Dissolving Microneedles Loaded with Lipid Nanocarriers for Vaginal Delivery of Clotrimazole: *In Vitro* and *Ex Vivo* Evaluation

**DOI:** 10.1021/acs.molpharmaceut.5c01721

**Published:** 2026-03-16

**Authors:** Paarkavi Udayakumar, Nataša Škalko-Basnet, Veronica Rondahl, Cristhian Fernando Salas Cotaquispe, Lisa Myrseth Hemmingsen, Georgios A. Sotiriou, Juan Du, Alexandra Teleki

**Affiliations:** † Department of Pharmacy, Science for Life Laboratory, 8097 Uppsala University, 751 23 Uppsala, Sweden; ‡ Department of Pharmacy, 8016 University of Tromsø The Arctic University of Norway, Universitetsveien 57, 9037 Tromsø, Norway; § Department of Animal Biosciences, Section for Anatomy, Physiology, Immunology and Pathology, 8095 Swedish University of Agricultural Sciences, 750 07 Uppsala, Sweden; ∥ Department of Microbiology, Tumor and Cell Biology, Karolinska Institute, 171 77 Stockholm, Sweden; ⊥ Department of Chemistry, Science for Life Laboratory, 7675 Stockholm University, 106 91 Stockholm, Sweden

**Keywords:** lipid-based formulation, liposome, poorly water-soluble drug, dissolvable microneedles, vaginal drug delivery

## Abstract

Vaginal yeast infections, such as vulvovaginal candidiasis (VVC), affect nearly three out of four women worldwide. Reoccurrence is frequent and requires repeated treatments with oral, antifungal medications at high doses. Prolonged treatments contribute to development of resistant fungal strains and the risk of systemic adverse effects. Vaginal drug delivery can overcome several of the disadvantages associated with oral drug administration. However, current dosage forms, such as vaginal creams and gels, are rapidly expelled from the vaginal tract and require daily dosing to ensure therapeutic outcome, thus jeopardizing patient compliance. Therefore, we developed rapidly dissolving microneedle arrays with a microneedle height reduction by 50% within 5 min, for local, vaginal delivery of antifungal drugs. Clotrimazole, a poorly water-soluble antifungal agent, was formulated in lipid-based nanocarriers (LNCs) and incorporated in the tips of microneedles. The antifungal activity was then tested against the most common VVC fungal strains, *C. albicans* and *C. glabrata*, using an *in vitro* disk diffusion assay and an explant model from bovine vaginal tissue. LNC loaded microneedles showed consistent significant inhibition of *Candida spp*. in comparison to blank microneedles and LNCs alone, with an inhibition diameter of 20–30 mm *in vitro* and a reduction of 3–5-fold fungal colonies *ex vivo*. Notably, the LNC-loaded microneedles inhibited fungal growth at a 10-fold lower drug dose than a commercial clotrimazole cream. Finally, a device prototype was developed in the form of an intravaginal ring incorporating multiple microneedle arrays on its surface, delivering a total drug dose of 0.1 mg per ring with 600 μm microneedle height. Local vaginal drug delivery using such microneedle-based devices could enable more effective treatment strategies for VVC.

## Graphical abstract

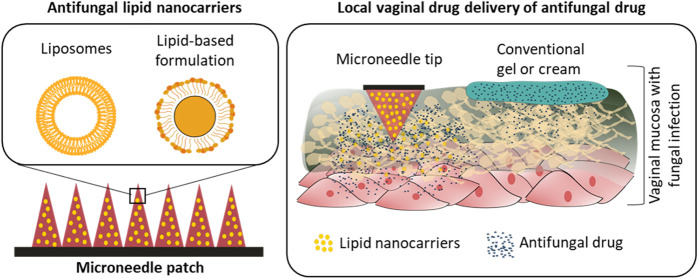

## 1 Introduction

During their lifetime, 70–75% of women acquire a vaginal fungal infection such as vulvovaginal candidiasis (VVC) and 50% of VVC patients suffer from relapse. The resulting chronic discomfort impacts sexual and reproductive health and quality of life. VVC is treated by oral administration of antifungal drugs, but is not without challenges regarding effective, local therapeutic outcomes.^1^ Higher doses of active pharmaceutical ingredients (APIs) are required to treat recurrent VVC, which increases the risk of systemic toxicity and has associated adverse effects that limit patient compliance. Furthermore, the long-term use of antifungal medication at high doses can contribute to the development of drug resistance.^2^ Clotrimazole, a first-generation antifungal, broad spectrum triazole, is commonly used to treat VVC. Current dosing regimens of clotrimazole include oral tablets at 100–200 mg daily, often administered in combination with fluconazole, another broad-spectrum antifungal agent.^3,4^

Unlike oral administration of antifungal APIs, vaginal delivery avoids the harsh gastrointestinal environment and the hepatic first-pass effect. This allows lower drug doses to be administered and reduces systemic exposure.^5,6^ The most commonly used vaginal dosage forms are semisolid formulations such as creams and gels. For example, oral treatment of VVC is commonly accompanied by topical administration of 1% clotrimazole creams in the vagina. These topical formulations require at least daily dosing to ensure therapeutic outcome and are also cleared rapidly^7^ from the intravaginal tract due to their low viscosity and the self-cleaning fluid discharge of the vagina. Sustained and uniform vaginal drug delivery is further challenged by the cervicovaginal mucus barrier that efficiently traps and hinders particulates and foreign pathogens from penetrating the epithelial surface. For local delivery to vaginal tissue, drug molecules must penetrate into and through the mucus mesh, and distribute uniformly into the underlying tissue at sufficiently high concentrations.

Cervicovaginal mucus is mainly comprised of water, mucin, and electrolytes. It also harbors the vaginal microbiota and thus directly influences susceptibility to microbial infections.^8^ The thickness of the mucosal barrier varies between 0.8–2.4 mm depending on the menstrual cycle stage and age.^8,9^ Thus, drug delivery to the vaginal cavity is influenced by physiological properties such as menstrual cycle variations, pre- and postmenopausal conditions, and interference from sexual intercourse.^10^ Numerous strategies, including mucoadhesion, mucopenetration, and pH-sensitive systems for prolonged drug release, have been developed to overcome these barriers by using polymeric nanoparticles, liposomes, dendrimers, and inorganic nanoparticles.^11–13^ For example, mucus-penetrating polymeric particles with a dense PEGylated surface can rapidly penetrate mucus and reach the deeper, unstirred layers thus increasing drug retention.^14^ These nanocarrier-based strategies, specifically for treatment of VVC, have been adopted for different types of APIs such as small molecules, phytochemicals, and probiotics.^15–17^

Despite these advances in nanocarrier-based strategies for vaginal drug delivery, the washout mechanism of the vaginal cavity remains a significant challenge for effective local treatment of VVC. Recently, the incorporation of advanced drug formulations into muco-penetrative devices such as intravaginal rings (IVRs) or microneedle arrays have been proposed.^18–20^ The latter, arrays of miniaturized needles on the micrometer scale, are designed to penetrate biological barriers and create a transport pathway enabling APIs to cross and target underlying tissues. To date, microneedles have shown substantial promise for transdermal drug delivery therapeutics such as insulin, with successful outcomes in preclinical and early phase clinical trials.^21^

Local drug delivery with microneedles is also being studied in other tissues such as the mouth and gastrointestinal tract. A recent study has reported use of microneedles for delivering nanosuspensions of an antiretroviral drug in vaginal tissue.^22^ First studies by McCrudden et al., reported successful application of microneedle arrays for intravaginal drug delivery, where they efficiently delivered nanosuspensions of an antiretroviral drug into vaginal tissue using dissolvable microarray patches. However, major concerns with microneedle technologies includes their limited drug loading capacity and scale-up.^23^ To meet clinical requirements, they must rely on highly potent drugs administered at low doses, frequent application schedules and/or large patch sizes.^24,25^ Two studies have explored local treatment of VVC with microneedles loaded with probiotics or nanocrystals of antifungal agents. The nanoparticle-based drug delivery systems were loaded onto the microneedles and tested *ex vivo*. Microneedles showed higher efficacy against the fungal biofilm, and a controlled drug release was obtained from the nanoparticles. Nevertheless, drug loading in microneedles remains a challenge, despite using nanocrystals that significantly increase dose delivery.^26,27^ Innovative devices that can deliver microneedles loaded with enabling drug formulations in the vaginal tract are needed to attain clinically efficient doses for VVC.

To this end, we developed rapidly dissolving microneedles loaded with lipid nanocarriers (LNCs) for clotrimazole as a localized treatment strategy for VVC. The selected LNCs were liposomes and a lipid-based formulation (LBF), as both of these carriers dissolve the highly lipophilic API clotrimazole (logP = 5) well.^28–31^ We developed a formulation strategy to incorporate the LNCs into the microneedle tips and studied their antifungal activity in relevant *in vitro* and *ex vivo* models for VVC. Finally, we created a prototype delivery device based on a 3D-printed intravaginal ring with its surface coated with multiple microneedle arrays. Our study demonstrates the potential of microneedle arrays loaded with LNCs to deliver APIs via the vaginal route for simple and effective treatment of vaginal infections such as VVC.

## 2 Materials and Methods

### 2.1 Materials

Labrasol ALF (Caprylocaproyl Polyoxyl-8 glycerides), Capryol 90 (Propylene Glycol Monocaprylate NF) and Labrafac lipophile WL 1349 (Medium chain triglycerides) were kindly provided by Gattefossé (Lyon, France). Lipoid S100 (>94% phosphatidylcholine) was provided by Lipoid GmbH, Ludwigshafen, Germany. Clotrimazole (≥99%), cellulose acetate filters (0.45 μm, Sartorius), and dialysis membrane (molecular weight cutoff 12–14 kDa) were obtained from VWR, Stockholm, Sweden. Sulforhodamine B, Tween 80, sodium acetate, ammonium acetate, acetonitrile (>99.9%), methanol (>99.9%), Mueller-Hinton agar, potato dextrose, glucose, agar, poly­(vinyl alcohol) (PVA; 87–89% hydrolyzed, MW 13–23 kDa, Sigma-Aldrich), poly methyl methacrylate (PMMA; MW ∼ 120 kDa, Sigma-Aldrich) and paraformaldehyde were obtained from Merck (Darmstadt, Germany). Canesten cream 1% (Bayer AB, Solna, Sweden) was procured from a local Swedish pharmacy and bovine vaginal tissue from a local abattoir (Lövsta kött AB, Uppsala, Sweden). The bovine vaginal tissues were obtained post-mortem from a licensed slaughterhouse after routine slaughter for the food industry and therefore considered as animal byproducts. According to Swedish regulations, the use of animal byproducts that was screened for infection by the licensed abattoir does not require ethical approval. Clinical isolates of *C. albicans* (U251) were obtained as samples from a previous study^22^ and *C. glabrata* (44136) was obtained from CCUG, Gothenburg, Sweden. The vaginal epithelial cell line VK2/E6E7 was obtained from ATCC, Teddington, UK. A cell viability assay kit (CellTiter-Fluor, G6081) was purchased from Promega (Nacka, Sweden). Techno Plastic Products AG tissue culture plates, 96-well plates, polyethylene glycol 400 and benzyl alcohol were purchased from Sigma-Aldrich, Solna, Sweden. Artificial cervicovaginal mucus was obtained from Bac^3^gel, Porto Salvo, Portugal. Calcofluor White and Concovalin Texas red were obtained from ThermoFisher, Uppsala, Sweden.

### 2.2 Preparation of Clotrimazole-Loaded LNCs

Liposomes (Figure 1a) were manufactured using the thin film hydration method.^31^ The liposomes were prepared using Lipoid S100, a phosphatidylcholine-based lipid excipient. Clotrimazole (30 mg) and Lipoid S100 (300 mg) were dissolved together in methanol and then placed in a rotary evaporator (IKA RV10 digital, IKA-Werke GmbH, Staufen, Germany) for 1 h at 60 mmHg and 45 °C to remove the solvent. The lipid film was hydrated using 10 mL Milli-Q water. The flask was shaken and vortexed gently until the film was dislodged to obtain the liposomal suspension. Size extrusion was performed by passing the suspension stepwise through 0.8, 0.4, and 0.2 μm filters (Nuclepore Track-Etch Membrane, Whatman House, Maidstone, UK). This was repeated five times at each step. The liposomes were stored at 4 °C overnight, afterwhich the liposomal suspension (3 mL) were dialyzed against 2 L of Milli-Q water for 4 h the next day and sink conditions were maintained, to separate free drug from the liposomes.

**Figure 1:**
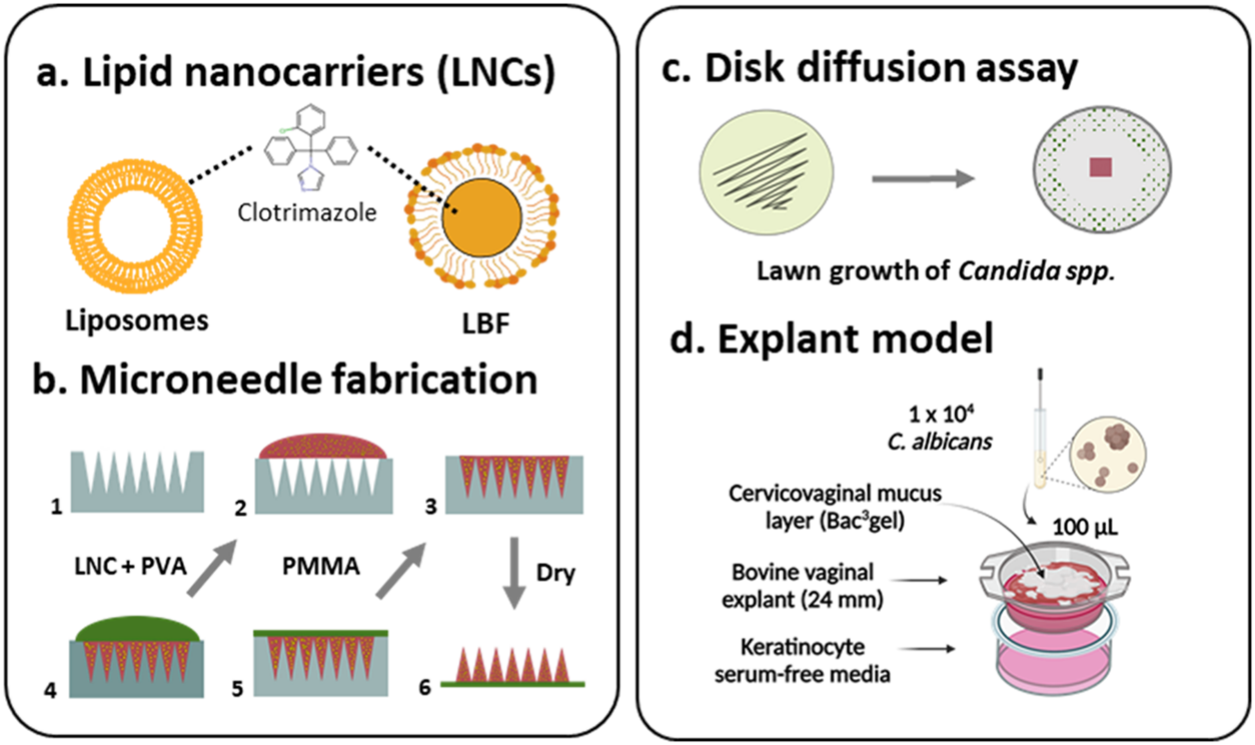
Schematic of the development of lipid nanocarrier (LNC)-loaded microneedle arrays and the *in vitro* and *ex vivo* characterization of their antifungal activity for treatment of vulvovaginal candidiasis (VVC). (a) Liposome and a lipid-based formulation (LBF) were selected as LNCs for the antifungal, active pharmaceutical ingredient (API) clotrimazole. (b) Flowchart visualizing the fabrication steps for LNC-loaded microneedle arrays. (Step 1): Microneedle molds with needle heights of either 600, 800, or 1000 μm were filled with (step 2): LNC-loaded water-soluble PVA and (step 3): the formulation (indicated in red) was deposited in the cavities by centrifugation and dried overnight. (Step 4): PMMA was then added to serve as a water-insoluble backing layer, shown in green. (Step 5): The PMMA layer was centrifuged and dried overnight. (Step 6): Finally, the microneedle arrays were removed from the mold. (c) Growth inhibition of *Candida species pluralis* (*Candida spp.*): *C. albicans* and *C. glabrata* was evaluated for LNCs and LNC-loaded microneedle arrays in an agar disk diffusion assay. (d) Their antifungal activity was also evaluated in an explant model with *C. albicans* infected bovine vaginal tissue and artificial cervicovaginal mucus layer (Bac^3^gel).

The lipid-based formulation (LBF, Figure 1a) for clotrimazole was adapted from literature.^29,30^ The composition and excipient ratios are detailed in Table S1. The continuous phase, comprising the surfactant Labrasol ALF and the cosurfactant Capryol 90, was prepared by weighing and vortexing the components for 10 s. Subsequently, the dispersed phase, comprising Labrafac lipophile, was added to the continuous phase and vortexed for an additional 10 s. Clotrimazole was then added to the lipid mixture and heated at 45 °C for 15 min with manual agitation every 5 min. The mixture was sonicated for 35 min at ambient temperature using a bath sonicator (ElmaUltrasonic S 80H, Singen, Germany) until a clear solution was obtained. Benzyl alcohol and propylene glycol were subsequently added as a preservative and a cosolvent, respectively (Table S1). A final sonication step was performed for an additional 15 min.

### 2.3 Characterization of Clotrimazole-Loaded LNCs

#### 2.3.1 Nanocarrier Size and Drug Loading

The hydrodynamic diameter and ζ-potential of the LNCs were determined by dynamic light scattering (DLS) and electrophoretic light scattering, respectively (Litesizer 500, Anton Paar GmbH, Graz, Austria). Liposomes were diluted 1:100 in Milli-Q water for DLS and LBFs were measured undiluted. Water and propylene glycol were selected in the solvent settings of the DLS software for liposomes and LBFs, respectively. The hydrodynamic diameter of the LNCs was monitored for up to 2 weeks at ambient temperature.

Clotrimazole quantification in LNCs was performed by high-performance liquid chromatography (HPLC; 1290 Infinity, Agilent Technologies, Santa Clara, CA) with a Zorbax Eclipse XDB-C18 column (4.6 × 150 mm, 3.5 μm) at 40 °C. LNCs were dissolved and diluted with methanol. The isocratic mobile phase was 75% acetonitrile and 25% sodium acetate buffer (25 mM, pH 5.0). Total run time was 8 min at a flow rate of 1 mL/min, resulting in a clotrimazole retention time of 4.2 min. The injection volume was 20 μL and UV absorbance was measured at 254 nm. Quality control samples were included for each analysis. A standard curve for clotrimazole in methanol was prepared over the range 2.5–25 μg/mL for liposomes and 50–100 μg/mL for LBFs, showing excellent linearity (*R* ^2^ = 0.9998 and 0.9997). The limit of detection (LoD) and limit of quantification (LoQ) were determined to be 0.5 and 1.5 μg/mL, respectively. For liposomes, the clotrimazole entrapment efficiency (EE, %) was calculated from the ratio of encapsulated clotrimazole over the amount available in the liposomal suspension after size extrusion (eq 1)­

1 $$\mathrm{EE}\%=\frac{\mathrm{amount of drug after dialysis}}{\mathrm{amount of drug before dialysis}}\times 100$$

In contrast, clotrimazole recovery was reported for the LBF, since no drug separation method was employed there. The drug content was quantified directly in the final formulation via HPLC and compared to the theoretical drug added during preparation.

#### 2.3.2 *In Vitro* and *Ex Vivo* Clotrimazole Release from LNCs

Clotrimazole release from the liposomes was determined in a Franz diffusion cell (Permegear, Inc. Hellertown, Pennsylvania). A 15 mm jacketed Franz cell was used, with 12 mL receptor volume and the system was maintained at 37 °C using a water bath. A semipermeable cellulose acetate membrane (*in vitro)* (pore size 0.45 μm, thickness 120 μm) and excised bovine vaginal tissue (*ex vivo)* (3 mm thick) was used as barrier between the donor and receiver compartments. The receiver compartment contained phosphate buffered saline (PBS) (1×, 0.01 M) with isopropyl alcohol as a cosolvent at a ratio of 1:1 to maintain sink conditions. Liposomes and LBF (200 μL) were added to the donor compartment. The sample ports were temporarily sealed with Parafilm throughout the experiment, to prevent evaporation of the solvent. Aliquots (500 μL) were taken from the receiver compartment at different time points (2, 4, 6, and 8 h) for *in vitro* and an additional 24 h tissue penetration time point for *ex vivo*. Aliquoted volumes were replaced with an equivalent volume of receiver medium. Cellulose membranes and tissue barrier were extracted for their drug retention using methanol. The clotrimazole content in the aliquots, membrane/tissue and donor compartment sample was quantified by HPLC using the previously described analysis method.

#### 2.3.3 *In Vitro* Biocompatibility of LNCs

The viability of vaginal epithelial cells (VK2/E6E7) after exposure to LNCs was determined using a fluorescence assay, CellTiter-Fluor. The cells were seeded at a density of 3 × 10^4^ cells/well on a 96-well tissue culture plate, and incubated for 24 h at 37 °C, 5% CO_2_ for adherence of the cell monolayer to the wells. Blank and clotrimazole-loaded LNCs that were diluted to normalize concentrations (10 μL LNCs per well, with a clotrimazole dose of 20 μg) were pipetted onto the cells and incubated for 24 h. Subsequently, the wells were washed with (1×, 0.01M) PBS and 100 μL of the CellTiter-Fluor reagent was added for incubation at 37 °C, 5% CO_2_ for 30 min. The fluorescence in the 96-well plate was analyzed with a microplate reader at 380–400 nm_Ex_/505 nm_Em_ (SpectraMax i3x, Molecular Devices, San Jose, CA).

### 2.4 Fabrication of Microneedle Arrays

Microneedle arrays with dissolvable, LNC-loaded tips were prepared following a simple molding process.^32^ Polydimethylsiloxane (PDMS) silicone molds (Micropoint technologies, Singapore) were used to fabricate microneedle arrays as a patch with tip heights of 600, 800, and 1000 μm (Figure 1b, step 1) with 10 × 10 tips per patch and a total patch area of 1, 1.5, and 2 cm^2^, respectively. To create the dissolvable microneedle tips for the arrays, poly­(vinyl alcohol) (PVA) in water (30% w/v) was mixed with liposomes or LBF at a 1:1 ratio. Sulforhodamine B (10 mg) was added to the PVA-LNC solution to enhance microscopic visualization of the microneedle tips. PVA-LNC (50–100 mg) was then added to the PDMS molds (step 2). The microneedle patches were centrifuged for 30 min at 1575 g (Eppendorf centrifuge 5810 R). Excess PVA-LNC was removed with a spatula and the molds were centrifuged a second time (30 min at 1575 g) to concentrate the LNC formulations in the microneedle tips (step 3). Subsequently, 100 mg of the water-insoluble backing layer composed of poly­(methyl methacrylate) (PMMA) in ethyl lactate was added to the PDMS silicone molds (step 4) and centrifuged for 30 min at 1575 g (step 5). The molds with the PVA-LNC tips and the PMMA backing layer were left to dry at 50 °C overnight and the microneedle patches were dislodged from the molds as a final product (step 6).

Microneedle tip morphology was visualized by optical (Nikon Eclipse Ts2R-FL, NY) and scanning electron microscopy (SEM; Zeiss 1550 AS02, LEO1550). The samples were sputter coated with an Au/Pd coater (Polaron SC7640) prior to SEM imaging. The drug load in the microneedle tips of different heights was quantified by HPLC using the previously described analysis method. For this, the microneedle tips were dissolved in a vial containing 2 mL water and placed in a bath sonicator for 15 min. Then, 2 mL methanol was added and the solution was centrifuged at 22000 *g* at ambient temperature for 10 min. Samples were analyzed using HPLC against a standard curve of the drug in methanol over the range 1.5–25 μg/mL.

To analyze the redispersion of the LNCs from microneedles, the microneedle arrays were dissolved in 1 mL Milli-Q for 20 min. The resulting solution was filtered using 100 kDa centrifugal filters (Vivaspin filters, Merck) at 4000 g for 10 min at room temperature to separate polymer components from the LNCs. The hydrodynamic diameter of both retentate and filtrate fractions was determined using DLS.

### 2.5 Insertion Capability of Microneedle Arrays in Bovine Vaginal Tissue

Microneedle arrays (*n* = 3) with a tip height of 1000 μm were inserted on bovine vaginal tissue for 30 s at ambient temperature, by using a texture analyzer (Stable Micro Systems, Surrey, UK) at a force of 3 N and a speed of 1 mm/sec. The microneedle tips were visualized after tissue insertion by optical microscopy and SEM. The vaginal tissue, post microneedle insertion, was snap-frozen in optimal cutting temperature (OCT) medium and cryo-sectioned (Cryostat NX50, Gothenburg, Sweden) with a slice thickness of 10 μm. The cross-sectional tissue slices were stained with methylene blue and imaged by optical microscopy (Nikon Eclipse Ts2R-FL, New York).

### 2.6 *In Vitro* Agar Diffusion Test of Antifungal Activity

A disk diffusion method was used to study the growth inhibition of *C. albicans* and *C. glabrata* by LNCs and LNC-loaded microneedle arrays (Figure 1c). Whatman disks (6 mm) were soaked with 10 μL of either clotrimazole-loaded LNCs, Canesten cream, or blank controls. Each drug-loaded disk corresponded to a dose of 20 μg of clotrimazole. The disks were incubated at 35 °C overnight. The microneedle arrays were applied on the agar plates as-prepared resulting in a variable drug loading across the different microneedle height. Mueller-Hinton agar with 2% glucose and 0.5 μg/mL methylene blue was used to obtain a semiconfluent lawn growth of *C. albicans* or *C. glabrata* following previously set guidelines for disk diffusion against yeasts.^33,34^ The LNC-loaded disks and microneedle arrays were applied on the agar plates within 15 min after fungal inoculation. The plates were then incubated at 35 °C for 24 h. Post incubation, the diameter of the inhibition zones was measured using a Vernier calliper.

### 2.7 *Ex Vivo* Antifungal Efficacy of LNCs and Microneedles

A vaginal explant model^35^ with a *C. albicans* biofilm on the mucosa was used to study the antifungal activity of the LNCs and microneedle (tip height = 1000 μm) arrays (Figure 1d). Excised bovine vaginal tissue was obtained within 1 h after slaughter and kept on ice for a few hours until preparation of the tissue explant. The explants (*n* = 24) were punched out (24 × 4 mm) using leather punches made of steel and were immediately washed twice with PBS (1×, 0.1 M) containing 50 mg/mL Gentamicin, as the standard 2 mg/mL of penicillin and streptomycin was not sufficient to eradicate the severe bacterial contamination of the tissue. The air–water interface between the explant and media was maintained using a transwell setup. The explants were suspended on the transwell plates with 2 mL serum-free keratinocyte media in the bottom of the plates. Keranocyte media was used for the vaginal epithelium as keratinocytes are predominantly present in the epidermis and the serum free factor of the media increases reproducibility without contamination. Artificial human cervicovaginal mucus (450 μL, Bac^3^gel) was pipetted on top of each explant, resulting in an average mucus thickness of 1 mm on the vaginal tissue. Subsequently, the explants were incubated at physiological conditions (37 °C, 5% CO_2_) for 24 h. Then *C. albicans* inoculum (1 × 10^6^ CFU (colony forming unit) /mL,) was added (100 μL) to the explants and incubated for 48 h to allow for the fungal biofilm to form. After the fungal incubation, 100 μL of LNCs per well (with a dose of 200 μg of clotrimazole) or the LNC-loaded microneedle arrays (as prepared with variable clotrimazole load depending on microneedle height) were applied on the explants.

After 24 h of treatment, the explants were dissected into smaller segments and used for different analyses: (i) CFU counting, (ii) confocal microscopy, (iii) SEM imaging and (iv) histological analysis. For CFU counting, the explants were cut into smaller bits with a scalpel and suspended in 5 mL PBS (1×, 0.1M), followed by tissue homogenization using a vortex. Serial dilutions were plated on yeast–potato–dextrose agar plates. The plates were incubated for 24 h, after which single colonies were counted. For confocal microscopy, tissue samples were immediately stained using Calcofluour (a stain for fungal hyphae) and concanavalin A-Texas red (a stain for the extracellular matrix) for 20 min at room temperature and subsequently imaged (Leica Stellaris 5 LIA, Leica Microsystems). Tissue samples for SEM imaging were fixed using 10% paraformaldehyde overnight and dehydrated in 20% ethanol for 1 min, followed by successive 1 min incubations in gradient concentrations of ethanol (40–100%) over a period of 8 min. The samples were stored in absolute ethanol and dried at room temperature for an hour before imaging (Aquilos 2 Cryo FIB, Thermofisher).

For histological analysis of the vaginal tissue after treatment with either LNCs or microneedles, the explants were fixed in 4% paraformaldehyde, dehydrated through a graded ethanol series (70 to 100%), and embedded in paraffin. Paraffin blocks were sectioned at a thickness of 5 μm, the sections were processed according to routine protocols and stained using hematoxylin and eosin (H&E) for histological evaluation and scoring to assess the effects of the LNC formulations and LNC-loaded microneedles on the fungal explant. The sections were scored by a veterinary anatomic pathologist (VR), blinded to sample treatments and without knowledge of the different treatments used. ANIKON Eclipse Ci microscope, an imaging Source DFK 33UX264 camera and the IC Capture 4 software was used for visualization, measurements and photography. Tissue lesions and fungal presence was scored according to Mann et al.,^36^ where 0 = with normal limits, 1 = minimal change i.e., the lesion barely exceeds that which is considered within normal limits, 2 = slight i.e., the lesion is easily identified but of limited severity, 3 = moderate i.e., the lesion is prominent but with significant potential for increased severity, and 4 = severe i.e., the lesion is as complete as possible and occupies the majority of the section or organ.

### 2.8 3D-Printed Intravaginal Ring with Microneedle Arrays

A prototype device for intravaginal delivery of the microneedle arrays was designed. The device was based on an intravaginal ring design (IVR; 56 × 7.6 mm) with cavities on the surface for placement of 20 microneedle arrays (7 mm^2^ each). The IVR was 3D-printed using an elastic methacrylate-based polymer by stereolithography (Formlabs Elastic 50A Resin V2, Somerville, MA). Microneedle arrays with a needle height of 600 μm were fitted and glued into the cavities of the IVR. The microneedle-coated IVR was evaluated for its insertion capability using a 3D-printed ring insertion model. The model consisted of a rigid outer cylinder from polylactic acid (outer diameter: 87 mm) and a soft inner lattice lining (thickness: 10 mm) fabricated from silicone 40A (Formlabs). The former was printed using fused deposition modeling and the latter was printed using stereolithography. A synthetic vaginal tissue lining (SynDaver) was placed on top of the inner lattice structure. The IVR prototype with 20 cavities was inserted into the tissue-lined model. Sixteen of the cavities were filled with microneedle arrays, while four were intentionally left empty to facilitate handling and insertion. The IVR was left in the ring model for 20 min, after which it was removed and the microneedle insertion capability was assessed by visual inspection of the tissue surface.

### 2.9 Statistical Analysis

Data are represented as the mean with standard deviation (SD). Student’s *t* test was performed to compare the means of two different formulations using GraphPad Prism 10.2.2. A *p*-value < 0.05 was considered significant.

## 3 Results and Discussion

### 3.1 Clotrimazole-Loaded LNCs

Clotrimazole is a broad-spectrum antimycotic drug commonly used to treat VVC. The local bioavailability and antifungal efficacy of clotrimazole is limited by its poor water solubility (0.49 μg/mL at pH 7.4 and 25 °C).^37,38^ Thus, it is commonly delivered in enabling drug formulations that can improve its solubility. Here we incorporated clotrimazole into two different lipid-based nanocarriers (LNCs): liposomes and a lipid-based formulation (LBF), both of which are suitable for local vaginal drug delivery.^29–31^ The as-prepared liposomes exhibited a hydrodynamic diameter of 170 ± 10 nm with a narrow size distribution, as indicated by a polydispersity index (PDI) < 0.2, and a ζ-potential of +16 mV. The lipid vesicles in the LBF were larger, with a hydrodynamic diameter of 345 ± 80 nm and a broader size distribution (PDI = 0.3). LIPOID S100 was used to make neutral liposomes, resulting in a ζ-potential close to zero. This is in agreement with literature for liposomes formulated with this lipid.^31^ Neutral liposomes generally exhibit greater stability in biological fluids and reduced electrostatic interaction with the mucus network, which facilitates improved diffusion through mucosal layers.^11^ ζ-potential measurements for the LBF could not be obtained due to turbidity even after dilution, thereby compromising accurate electrophoretic measurements. Formulations with hydrodynamic diameters <500 nm are more effectively transported through the cervicovaginal mucus barrier than larger ones.^8^ The clotrimazole-loaded LNCs manufactured here were thus in a size range suitable for overcoming the mucus barrier for local vaginal drug delivery. The morphology of both liposomes and LBF has previously been characterized by electron microscopy, where spherical vesicular structures were observed.^30,31^

Both LNCs exhibited good physical stability during storage at ambient temperature for up to 2 weeks as the droplet sizes did not significantly increase (Table S2). Specifically, for the LBF, the storage stability was enhanced by adding benzyl alcohol to the formulation, as droplet size had increased without it (data not shown). The LBF solubilized clotrimazole 20 times more than the liposomes. The clotrimazole drug load was 35 ± 2.3 mg/g and 1.5 ± 0.25 mg/mL in LBF and liposomes, respectively (Table S3). Clotrimazole recovery in LBF was 70% and clotrimazole recovery and entrapment efficiency in liposomes was 75 and 90% respectively. No drug precipitation was seen in either LNCs during the two-week storage period and the drug content remained unchanged in the formulations. The clotrimazole load in the LNCs achieved here is in line with the literature.^30,31^ In liposomes, the lipophilic clotrimazole is likely located in the lipid bilayer (Figure 1a) and the drug loading capacity there is limited due to the small phospholipid bilayer volume compared to the lipid droplets in the LBF. However, liposomal vesicles <200 nm, as produced here, are preferred for their higher physical stability compared to larger ones. In the LBF, the mixture of lipid (Labrafac lipophile), cosolvent (propylene glycol) and surfactants (Labrasol ALF and Capryol 90) aids in the solubilization of clotrimazole and thus a significantly higher drug load can be achieved compared to the liposomes.

The biocompatibility of the blank and clotrimazole-loaded LNCs was evaluated *in vitro* against vaginal epithelial cells (Figure 2a). Cell viability was unaffected by the liposome formulations. In contrast, exposure to LBF reduced cell viability by 85%. This cytotoxicity can primarily be attributed to the excipients, as no significant difference in cell viability occurred between the blank and the clotrimazole-loaded LBF. The reduced cell viability associated with the LBF is likely related to the surfactant components (Labrasol ALF and Capryol 90), which are known to compromise cell membrane integrity at higher concentrations. However, since the individual excipients were not evaluated separately in this study, the effect cannot be conclusively assigned to a specific component. Importantly, *in vivo* studies investigating dermal and oral administration of this LBF have not reported adverse effects.^30,39^ The *in vitro* cytotoxicity observed in vaginal epithelial cells may therefore be overestimated, as 2D cell culture models do not fully recapitulate the complexity of the *in vivo* environment. Further *in vivo* investigations are warranted to comprehensively assess the biocompatibility of these formulations for vaginal drug delivery.

**Figure 2:**
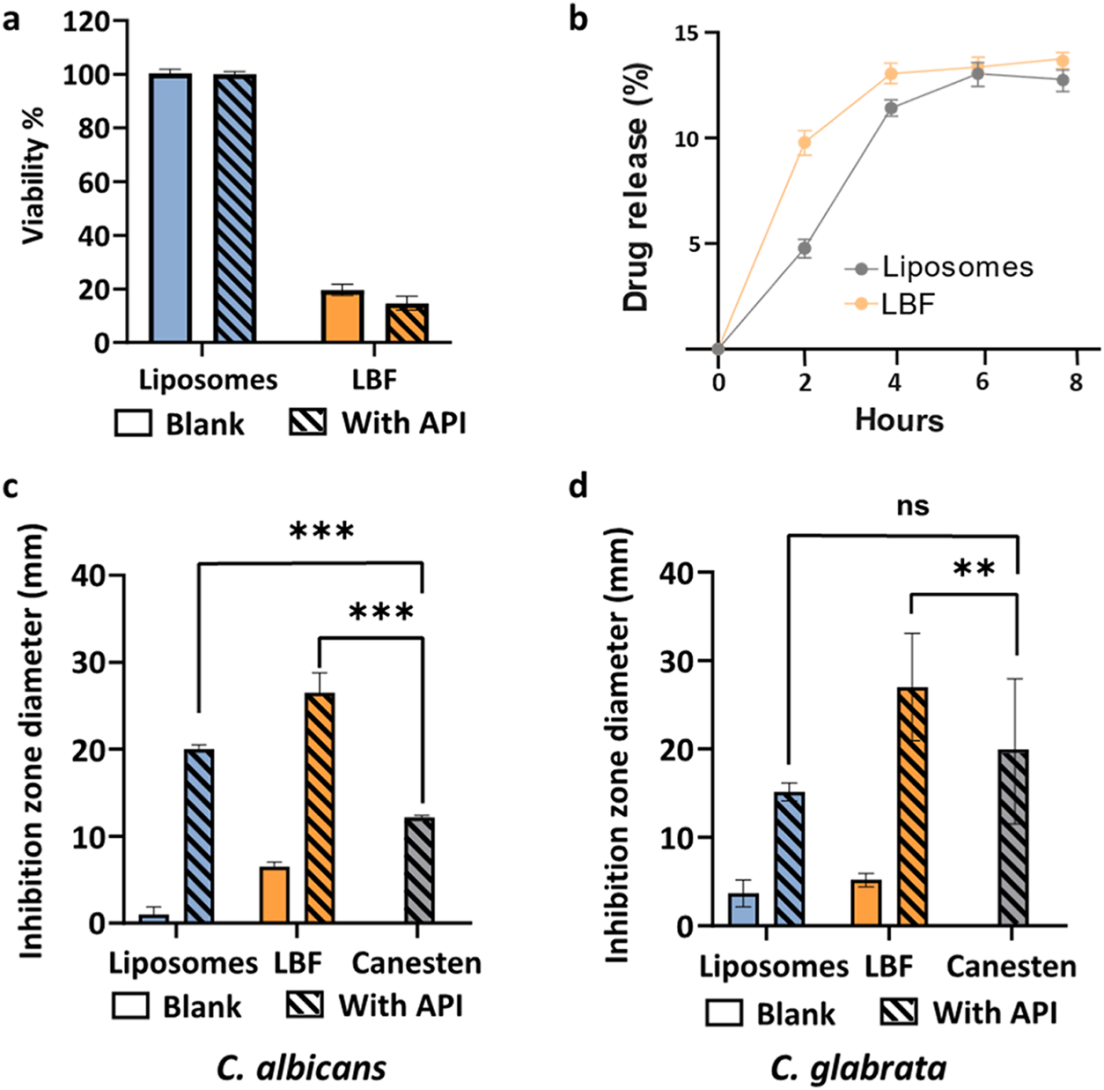
*In vitro* characterization of the toxicity, drug release profiles, and antifungal activity of LNCs. (a) Viability of VK2/E6E7 epithelial vaginal cells after 24 h of incubation with blank (solid fill) and clotrimazole-loaded (dashed lines) LNC formulations. (b) Drug release of clotrimazole from liposome (gray) and LBF (yellow) across a cellulose acetate membrane in a vertical Franz diffusion cell. Inhibition zone diameter in the agar disk diffusion assay against (c) *C. albicans* and (d) *C. glabrata* using blank and clotrimazole-loaded LNC formulations and commercially available Canesten cream with clotrimazole as the API. Data represent the mean of *n* = 3 ± standard deviation. *p* < 0.05 (*), *p* < 0.005 (**), *p* < 0.0005 (***) and ns >0.05.

Release of clotrimazole from the liposomes over time was measured *in vitro* in a Franz diffusion cell. To maintain sink conditions and prevent flux of aqueous buffer from the receiver to the donor compartment, a 1:1 mixture of PBS and isopropyl alcohol was used in the receiver compartment. This cosolvent system was selected based on the solubility profile of clotrimazole, which is highly soluble in alcohol (9 mg/mL in isopropyl alcohol at 25 °C)^40^ but exhibits poor solubility in water (0.49 μg/mL at pH 7.4 and 25 °C).^28^ Clotrimazole-loaded liposomes and LBF demonstrated a slow and sustained release, with 13% (3.2 mg) and 12.2% (1.6 mg) of the encapsulated drug, respectively, detected in the receiver compartment after 8 h (Figures 2b, S1). The cumulative drug release per unit area (1.77 cm^2^) over 8 h was calculated (Figure S2). The steady-state flux values for liposomes and LBF were 0.084 and 1.2938 mg·cm^–2^·h^–1^, respectively. While liposomes exhibited a linear release profile over the investigated time frame, the LBF displayed a nonlinear release behavior. Consequently, the flux for the LBF was calculated only over the initial linear phase (0–2 h).

The observed nonlinearity is likely attributable to formulation depletion and sink-driven destabilization of the lipid carrier in the receiver medium, rather than membrane-controlled diffusion. Even under sink conditions for free drug, complete release from lipid-based carriers may not occur, as highly lipophilic drugs can thermodynamically favor retention within the lipid phase.^41^ In addition, heterogeneous drug distribution within the formulation can limit release to the more accessible drug fraction, resulting in an apparent release plateau (∼15%). Together, these effects reflect intrinsic drug–lipid partitioning and system equilibrium rather than a limitation of the release methodology.^42,43^ Further investigation is therefore required to optimize the release characteristics of the LBF. Future studies will incorporate blank formulation in the receiver chamber to maintain formulation equilibrium and enable more accurate determination of drug flux.

*Ex vivo* drug release was analysed using excised bovine vaginal tissue on the Franz cell diffusion with the similar set up as mentioned before, where the donor samples, receiver aliquots and tissue were analyzed for their drug content after 24 h of drug release. Most of the drug (∼60%) was retained in the tissue after 24 h for both LNCs, and the liposomes had a slightly higher percentage of the drug that was permeated into the receiver media than LBF. About 25 and 40% of the drug was found to be in the donor chamber post 24 h for liposomes and LBF respectively (Figure S3).

In summary, both LNCs exhibited hydrodynamic diameters suitable for local vaginal drug delivery and good physical and chemical storage stability at ambient temperature for 2 weeks. Higher clotrimazole drug load could be achieved in the LBF compared to the liposomes. A higher drug loading capacity of the LNC is advantageous for its subsequent incorporation in microneedles, which have an inherently low drug-loading capacity. However, excipients used in LBFs, in particular surfactants, raise concerns regarding their biocompatibility and more detailed *in vivo* studies are warranted to ensure their safe use. Based on our *in vitro* data with epithelial vaginal cells, the liposomal clotrimazole formulation is thus advantageous in terms of its high biocompatibility.

### 3.2 *In Vitro* Antifungal Activity of LNCs

The antifungal activity of clotrimazole-loaded LNCs was tested against *C. albicans* (Figure 2c) and *C. glabrata* (Figure 2d) *in vitro* using a disk diffusion assay. Among the *Candida* species present during VVC infection, *C. albicans* (68%) and *C. glabrata* (19.2%) are the most abundant.^44^ *C. glabrata* tends to develop azole resistance and typically requires longer treatments at higher API doses.^33^ Growth inhibition was observed for all clotrimazole-loaded formulations against both fungal species. Notably, the inhibition zones against *C. albicans*, created by both liposome- and LBF-loaded disks, were significantly larger than for the commercially available clotrimazole cream (Figure 2c). This could be attributed to the greater diffusivity of the LNCs compared to the Canesten cream in the agar gels. For liposomes, the fungal growth inhibition could be primarily attributed to the API, as no effect was seen in the blank formulation. In contrast, also the excipients used in the LBF inhibited fungal growth to some extent as observed from the inhibition zones created by blank LBF. This observation is also in line with the cytotoxicity observed for the blank LBF (Figure 2a). LBF showed similar levels of inhibition against both *C. glabrata* and *C. albicans*. In contrast, liposomes exhibited a lower level of inhibition against *C. glabrata*, though comparable to Canesten cream. *In vitro* evaluation of the antifungal activity of the LNCs against two *Candida spp.* strains yielded inhibition zone diameters comparable to those reported in the literature, although *Candida albicans* is typically the primary strain used for initial testing of novel lipid nanoparticle formulations.^45–47^

### 3.3 LNC-Loaded Microneedle Arrays

In the next step, the incorporation of LNCs in dissolving microneedles was investigated, to circumvent rapid washout of formulations from the vaginal tract. The underlying hypothesis was that microneedles could enhance mucus penetration and prolong the contact time of the formulations with the vaginal mucosa. Microneedles with three different needle heights (600, 800, and 1000 μm) were fabricated via molding and the PVA-based tips were loaded with the LNC formulations. As expected, the drug load increased with increasing needle height and was higher for LBFs compared to liposomes, in agreement with the clotrimazole loading capacity of the formulations (Figure 3a, Table S3). Although the higher microneedle height is advantageous due to its capacity to hold a higher drug dose, there could be concerns regarding its safety in the vaginal tract. Previous studies have used 600 μm microneedles for vaginal drug delivery.^27,48^ The average vaginal mucosal thickness is clinically reported to be 0.8–2.4 mm.^9^ Hence, we postulate that a patch with 1000 μm needles could penetrate the mucus barrier at variable physiological thicknesses without reaching nerve endings that are usually found in the lamina propria and below.

**Figure 3:**
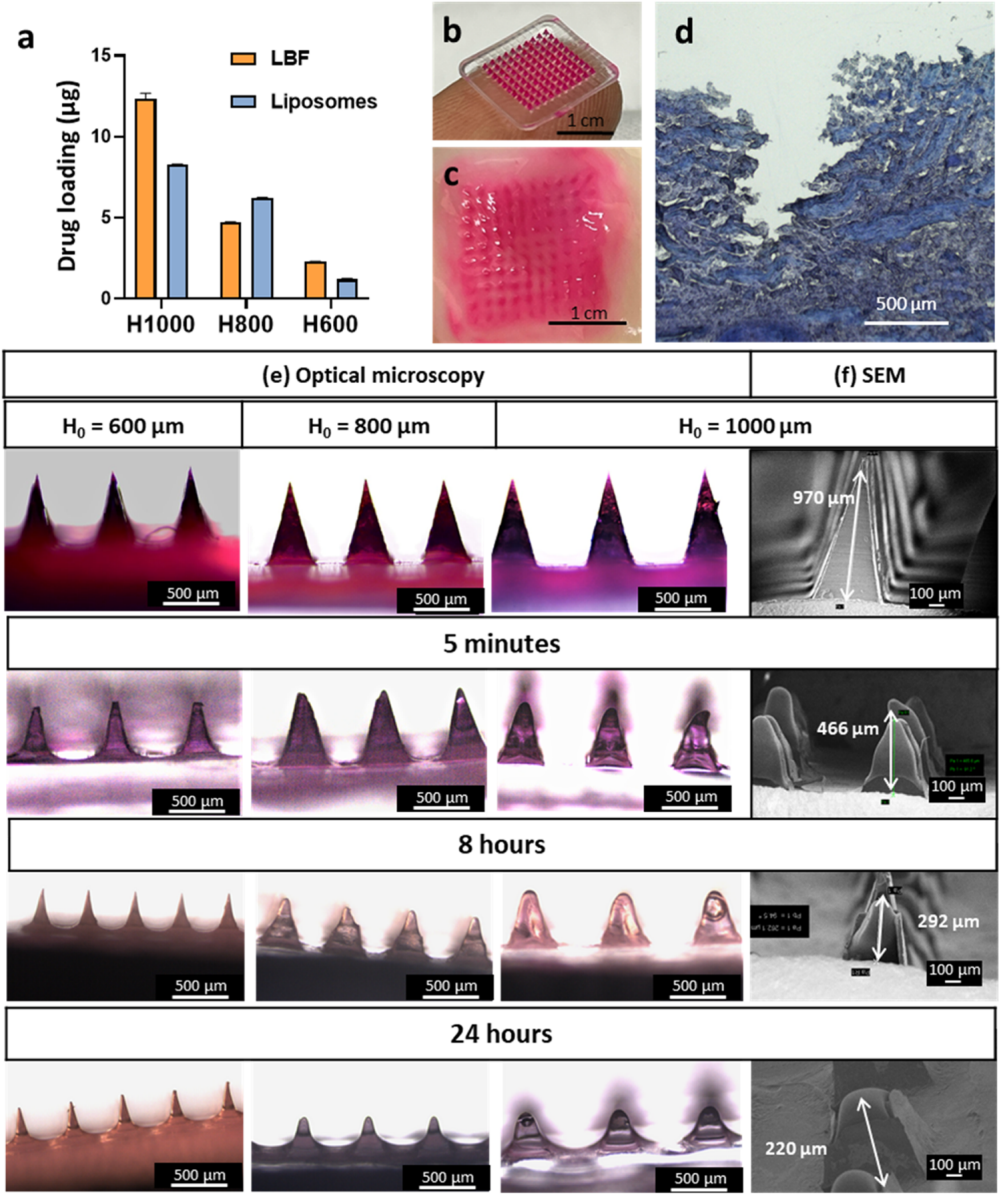
Microneedle morphology and insertion tests in bovine vaginal tissue. (a) Microneedle arrays with needle heights (H) of 600, 800, and 1000 μm containing clotrimazole-loaded liposomes (blue) or LBFs (orange). (b) Digital image of the liposome-loaded microneedle patch (H = 1000 μm) with an area of 2 cm^2^. The water-soluble LNC-loaded PVA in the microneedle tips was stained with a red dye for visualization. (c) Top-view, digital image of bovine vaginal tissue after insertion of the liposome-loaded microneedle patch. The image shows the holes created and the LNC deposition. (d) Bright field micrograph of vaginal tissue cross-section after microneedle insertion. Optical microscopy (e) and scanning electron microscopy (f) images of the liposome-loaded microneedle arrays with H = 600, 800, and 1000 μm as-prepared and after 5 min, 8 and 24 h insertion in the bovine vaginal tissue.

The dissolution of microneedle patches (Figure 3b) was tested in excised bovine vaginal tissue. The microneedles were inserted in the tissue at a controlled force of 3N, optimized to replicate the force that could be applied by a thumb. Thereafter the tips rapidly started dissolving within 5 min. The 10 × 10 pores created by the microneedle array and the Sulforhodamine B dye deposited there could clearly be distinguished with the naked eye in the tissue (Figure 3c). The tissue cross-section displayed a micropore ∼ 900 μm in depth and was thus in good agreement with the applied microneedle tip height of 1000 μm (Figure 3d).

Microneedle morphology before and after dissolution (up to 24 h) was evaluated using optical and scanning electron microscopy across different microneedle heights (Figures 3e,f, S4 and Table S4). The LNC-loaded microneedles exhibited sharp, well-defined, pyramid-shaped tips at all heights, indicating that LNC incorporation did not compromise microneedle fabrication. Following tissue insertion, a rapid reduction in tip height, approximately 50% within 5 min, was observed, attributed to the dissolution of the PVA-based tips upon contact with vaginal tissue. This rapid dissolution behavior is consistent with prior reports using PVA microneedles for transdermal drug delivery.^48,49^ At extended time points (8 and 24 h), a slower dissolution phase was noted following the initial rapid tip loss. This biphasic dissolution behavior could be attributed to partial extension of the PMMA backing layer into the microneedle tips, contributing to the remaining structure.^32^ Further stability characterization of the microneedles would be valuable in future studies. In particular, assessment of moisture uptake of the dissolvable tips and water vapor transmission properties of the LNC-loaded microneedles over extended storage periods would provide important insight into their long-term stability and performance.^17^

LNCs redispersed in Milli-Q water were analyzed by DLS to determine their hydrodynamic diameter. Both liposomes and LBFs maintained nanometer-scale particle sizes, with mean diameters of 210 ± 50 nm and 320 ± 80 nm, respectively. In contrast, the microneedle filtrate showed no detectable autocorrelation function and a high polydispersity index, indicating absence of measurable nanoparticles. Overall, these results demonstrate good redispersibility of the LNCs following microneedle tip dissolution.

### 3.4 *In Vitro* Antifungal Activity of LNC-Loaded Microneedles

The antifungal activity of the LNC-loaded microneedles was assessed using the *in vitro Candida spp.* disk diffusion assay (Figure 4) A significantly higher inhibition zone diameter against *C. albicans* was measured for the LNC-loaded microneedles (H = 1000 μm) compared to Canesten cream, despite the lower clotrimazole dose delivered with the microneedle arrays (LBF-loaded H1000 microneedle: 12.5 μg of clotrimazole/patch, liposome-loaded H1000 microneedle: 8.5 μg of clotrimazole/patch) compared to the commercial cream (20 μg of clotrimazole/disc) (Figure 4a,b). The antifungal activities of the 600 and 800 μm tips (see Figure 3a for drug loading of H600 and H800 patches) were also tested against *C. albicans*, with a lower inhibition zone diameter observed especially for 600 μm ones (Figure S5). This is in line with their low drug load (2–9 μg/patch, Figure 3a). In contrast, the inhibition zone diameters against *C. glabrata* were comparable for the microneedles and Canesten cream, and overall lower than for *C. albicans* (Figure 4c). This could be attributed to differences in the biofilm morphologies of *C. albicans* and *C. glabrata* and the more resistive nature of *C. glabrata*.^33,50,51^ Relevant *in vitro* inhibition zone diameters against *C. albicans* have previously been reported for microneedle-based antifungal systems. Nasiri *et al.* demonstrated inhibition zones of up to 68 mm using Amphotericin B nanoemulsion-loaded microneedles developed for dermal candidiasis.^47^ In that study, both the microneedle tips and the dissolvable backing layer contained the nanocarrier, resulting in a substantially higher total drug load per patch (427 μg). In contrast, in the present study, the drug was confined to the dissolvable microneedle tips only, leading to a lower overall drug content per patch.

**Figure 4:**
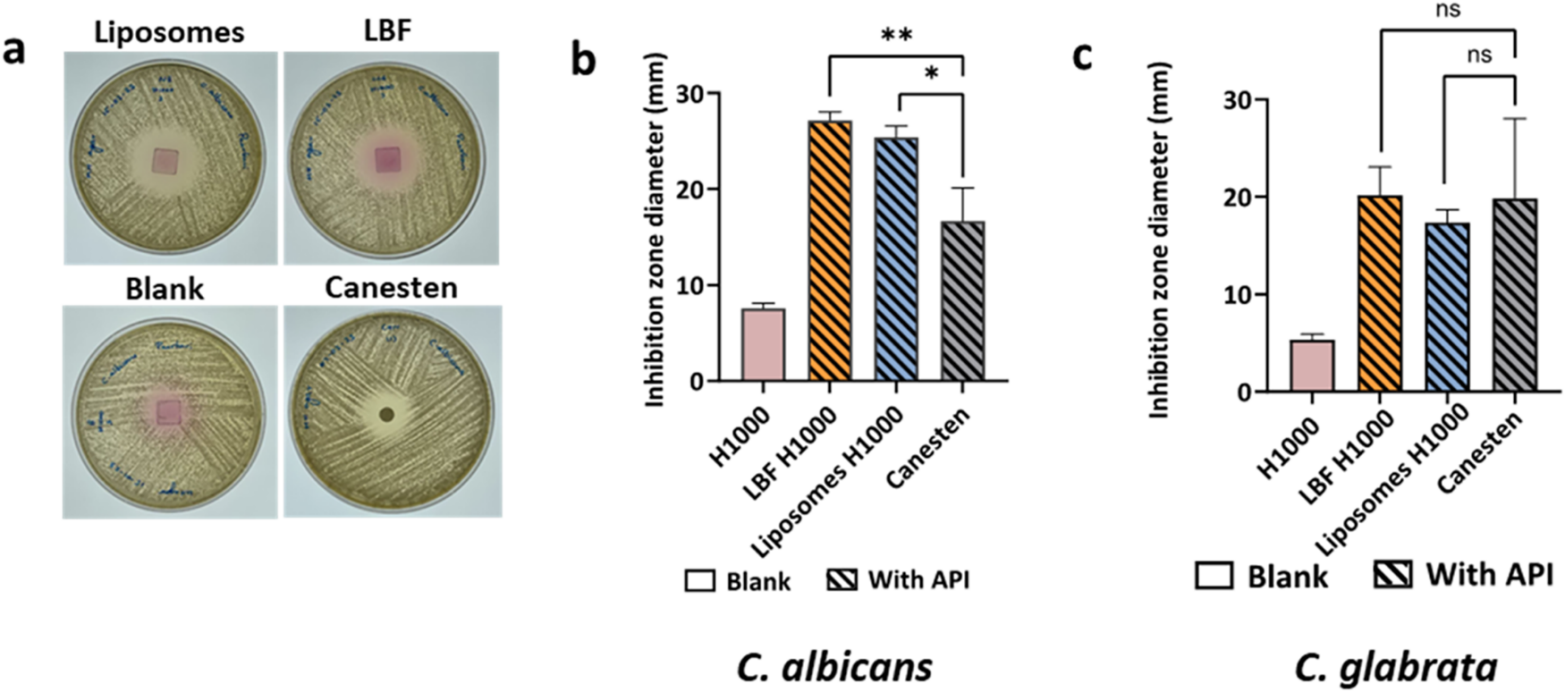
*In vitro* antifungal activity of LNC-loaded microneedle arrays (H = 1000 μm). (a) Optical images of the growth inhibition of *C. albicans* on agar plates after 24 h of treatment with Canesten cream, blank microneedles, and liposome- and LBF-loaded microneedles. Inhibition zone diameters in the agar disk diffusion assay against (b) *C. albicans* and (c) *C. glabrata* using blank microneedles, LNC-loaded microneedles, and Canesten cream. *p* < 0.05 (*), *p* < 0.005 (**), *p* < 0.0005 (***) and ns >0.05.

### 3.5 *Ex Vivo* Antifungal Efficacy of LNCs and LNC-Loaded Microneedles

Following the disk diffusion assay, the antifungal activity of the clotrimazole-loaded LNCs and LNC-loaded microneedles was further investigated using a more biorelevant assay. A bovine vaginal explant model infected with *C. albicans* was employed, with an artificial mucus layer applied on the tissue (Figure 1d). Figure 5a shows the timeline of inoculation and treatment with LNCs and LNC-loaded microneedles on the explant. Figure 5b shows confocal and SEM images of the noninfected and *C. albicans* infected explants. The extracellular matrix of the vaginal tissue is clearly visible in the noninfected explant. After *C. albicans* infection, fungal hyphae and buds are evenly distributed as a biofilm on the vaginal tissue. Morphological changes such as flaccid hyphae were evident in the fungal explants after treatment with clotrimazole-loaded LNCs (Figure 5b) and Canesten cream (Figure S6). However, Canesten cream did not spread out on the explant surface like the LNCs. This indicates that the LNCs diffused to a greater extent, in line with their small hydrodynamic diameter (Table S2).

**Figure 5:**
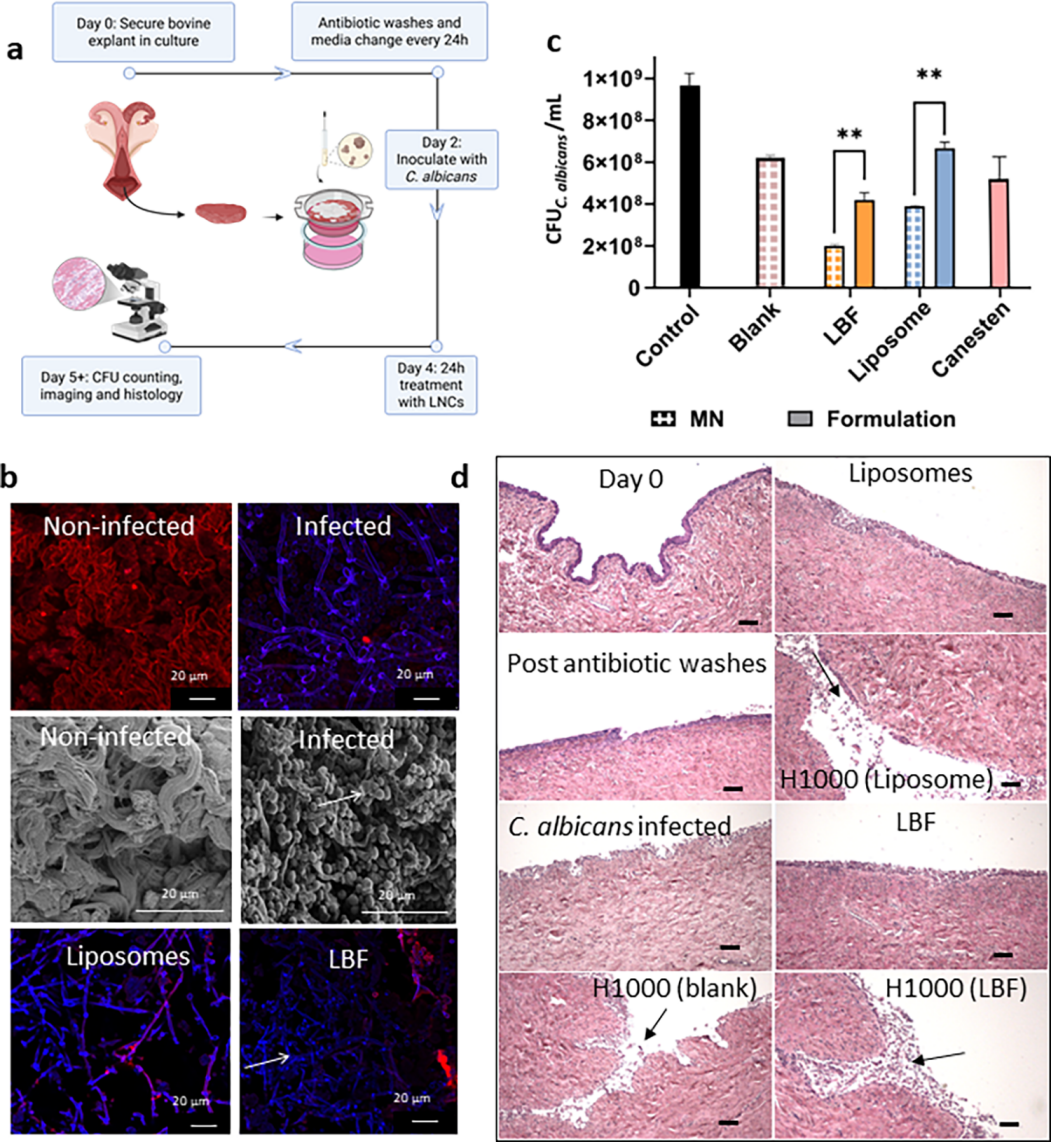
*Ex vivo* antifungal efficacy of LNC-loaded microneedles. (a) Timeline of the explant culture, infection and treatment. (b) Morphological features of the bovine vaginal explant model infected with *C. albicans* including confocal and scanning electron microscopy images of the vaginal tissue. (c) *C. albicans* CFUs after treatment of the explant model with LNCs, LNC-loaded microneedles or Canesten cream for 24 h. (d) Representative H&E stained vaginal tissue sections from the explant model before and after treatment with LNCs, blank microneedles and LNC-loaded microneedles, arrows pointing at micropores. Scale bars in (d) represent 100 μm.

The number of visible fungal colonies (CFU_*C. albicans*_) was quantified before and after treatment with blank and clotrimazole-loaded LNCs, and Canesten cream, compared to the control (Figure S7). Given that the *ex vivo* bovine vaginal explant model represents a densely infected and structurally complex tissue environment, a higher clotrimazole dose was employed (200 μg) compared to the *in vitro* agar disc diffusion assay (20 μg). In such tissues, drug penetration is hindered by extracellular matrix components, tissue architecture, and biofilm formation, necessitating a higher applied drug dose to achieve comparable antifungal effects. A significant reduction in CFU_*C. albicans*_ was obtained after treatment with clotrimazole-loaded LNCs and for Canesten cream, while the blank LNCs had no significant impact. The antifungal activity of clotrimazole-loaded formulations was thus comparable to Canesten cream in the explant model (Figure S7), in contrast to the significant differences between LNCs and Canesten observed in the disk diffusion assay (Figure 2c), highlighting the importance of assessing formulations in different preclinical models. Previous studies have demonstrated a good correlation between vaginal explant models and corresponding *in vivo* data.^48,52,53^ Overall, both liposomes and LBFs proved to be efficient delivery systems for clotrimazole in the treatment of vaginal fungal infections with an efficacy comparable to the commercial cream formulation (Canesten) of the API. However, when delivering these formulations locally in the vaginal tract, the washout mechanism of the vaginal tract reduces their efficacy.

To address this limitation, the LNCs were further incorporated into microneedles. When comparing the antifungal activity of the LNC-loaded microneedles with LNCs alone, a significant reduction in CFU_*C. albicans*_ was observed for microneedles (both blank and LNC-loaded) and Canesten cream compared to control (Figure 5c). The significant reduction in CFU_*C. albicans*_ with the blank microneedles, consistent with the disk diffusion assay results (Figure 4b), together with the enhanced fungal killing activated by microneedles at a lower drug dose compared to Canesten cream, suggest that microneedle penetration mechanically disrupts the fungal biofilm. This disruption likely increases the sensitivity of the biofilm to the antifungal API.^37^ This is supported by the growth inhibition obtained also for the blank microneedles *in vitro* and *ex vivo* (Figures 4a–c, 5c), as PVA itself is not expected to exhibit antifungal activity. The PVA from the microneedle tips exhibited a pH of 6.5 when dissolved in Milli-Q water, which could potentially alter the vaginal microenvironment and influence the growth of *C. albicans*. To assess this, growth was tested in two media with different pH values: acidic (acetate buffer, 25 mM, pH 4.6) and neutral/basic (PBS 1X. 0.01M, pH 7). Neither condition affected the growth of *C. albicans* when applied to an even lawn on agar plates. Blank microneedles of all three heights resulted in a comparable reduction in CFU_*C. albicans*_ (Figure S8) compared to the control. Thus, already a 600 μm tip height is sufficient to damage the fungal biofilm.

Most importantly, compared to the control, LBF-loaded microneedles reduced the CFU_*C. albicans*_ by 80%, which is significantly more than Canesten cream (50%), despite the lower drug load (Figure 3a, H1000:12.5 μg for the LBF-loaded microneedles vs 200 μg for Canesten). Liposome-loaded microneedles achieved a 60% CFU_*C. albicans*_ reduction, which is higher than Canesten cream, though not significantly. This is remarkable in light of the 10-fold lower clotrimazole dose delivered from the microneedles (Figure 3a, H1000:8.5 μg for the liposome-loaded microneedles vs 200 μg for Canesten). Finally, it is important to highlight that the CFU_*C. albicans*_ reduction was significantly higher for LNC-loaded microneedles treatment than the LNC formulations alone (Figure 5c). This demonstrates the capacity of microneedles to penetrate the mucus barrier, dissolve and release the drug formulations in close proximity to the vaginal mucosa and disrupt fungal biofilms, thereby lowering the therapeutic dose required for fungal growth inhibition. In the context of reported local antifungal delivery strategies, the efficacy achieved with the LNC-loaded microneedles falls within the range observed for other advanced systems evaluated against *Candida albicans.* The approximately 1-log reduction in fungal burden is comparable to reductions reported for fluconazole nanocrystal-loaded microneedles and probiotic (*Lactobacillus plantarum*) particle-based systems tested in *ex vivo* candidiasis models.^17,27^ Although these platforms rely on distinct mechanisms, enhanced drug loading within microneedle matrices or biological antagonism by probiotic components, they converge on similar levels of fungal reduction under *ex vivo* conditions. Importantly, the LNC-loaded microneedle platform offers potential advantages in dose sparing and improved mucosal penetration, which may facilitate sustained local exposure while minimizing off-target effects. Collectively, these findings indicate that the antifungal performance of the LNC-loaded microneedles aligns with the current state of advanced local delivery strategies for VVC, while underscoring the challenges associated with achieving complete eradication in complex mucosal infection models. Future studies should evaluate the efficacy of this microneedle-based delivery system against polymicrobial and mature *Candida spp.* biofilms to further assess its performance in more complex infection models.^54^

Histological analysis was performed on the explants treated with LNCs and LNC-loaded microneedles by examining cross-sectional H&E stained sections and comparing them with untreated controls. In explants subjected to antibiotic washing before and after *C. albicans* infection, disruption of the vaginal epithelium was evident (Figure 5d). On the other hand, LNC treated explants exhibited a rather intact vaginal epithelium compared to the infected control. Insertion of blank microneedles resulted in the formation of a micropore accompanied by epithelial disruption, and a comparable level of disruption was observed in explants treated with LNC-loaded microneedles (Figure 5d). The stained sections were subsequently evaluated through blinded histopathological scoring, assessing epithelial disruption, inflammation and fungal presence from the epithelial surface down to the muscualris layer of the explants (Figure 6 and Table S5). Histopathological assessment indicated minimal differences in the cytotoxicity between the two LNC formulations, in contrast to the *in vitro* findings (Figure 2a). Epithelial disruption includes attenuation, erosion and ulceration of the epithelium, and extent of epithelial single cell death (Table S5). The degree of epithelial disruption associated with microneedle insertion was consistent with expected mechanical penetration effects (Figure 3d). Across all explants, epithelial disruption was consistently more severe in fungal-inoculated controls (Figure 6c), lipid nanocarrier formulations (Figure 6d,e), and microneedle-treated groups (Figure 6f–h), which had severely attenuated and eroded epithelium with moderate to severe single cell death, than in baseline (Day 0) and negative controls (Figure 6a,b), which had minimal-to-moderate disruptions (Figure 6 and Table S5). The majority of interventions, including slaughterhouse handling, explant punching, antibiotic washing, transfer to transwell culture, fungal inoculation, and subsequent treatments contributed to substantial epithelial loss and cellular death, further compounded by necrosis of the tissue over time. Owing to the inherent limitations of the *ex vivo* model, epithelial regeneration and repair could not be evaluated and will require confirmation in an *in vivo* setting.

**Figure 6:**
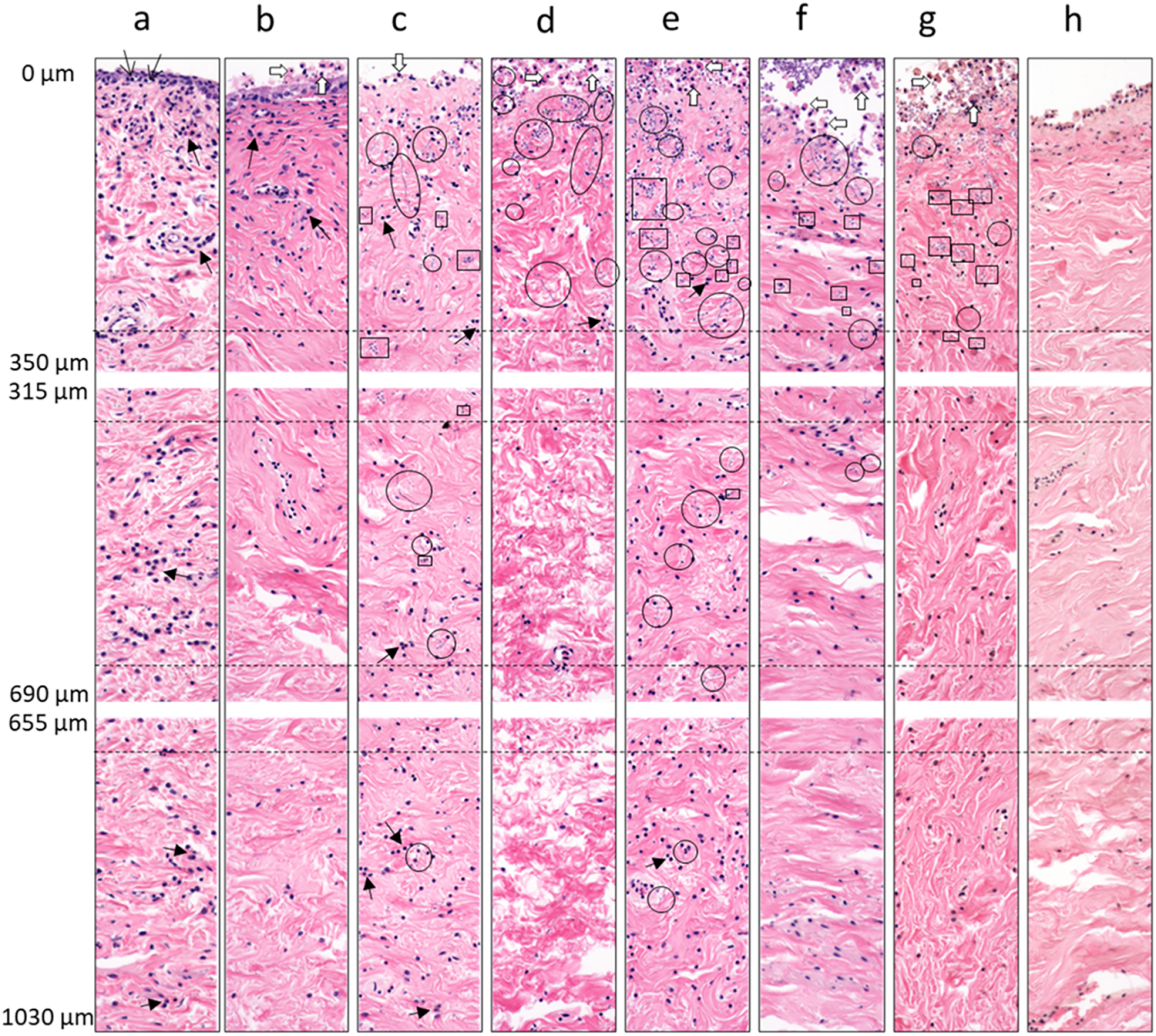
Representative histological cut-outs highlighting key features used for epithelial damage scoring. Treatment groups: (a) Day 0 explant, (b) negative control (uninoculated), (c) positive control (fungal-inoculated), (d) liposome-treated, (e) lipid-based formulation (LBF)-treated, and (f–h) blank microneedle patches (H1000, H800, H600). Circled areas indicate fungal hyphae; square areas indicate yeast bodies; thin black arrows denote intraepithelial lymphocytes; black arrows with broad arrowheads indicate small inflammatory cell aggregates (mainly lymphocytes); white broad arrows denote single-cell epithelial death.

### 3.6 IVR Prototype for Delivery of Multiple Microneedle Arrays

An IVR prototype was designed with a surface decorated with multiple LNC-loaded microneedle arrays. Marketed IVRs like the NuvaRing, have an outer diameter of 56 mm, and a cross-sectional diameter 4–8 mm. Hence, we fabricated a polygonal IVR by stereolithography, 56 mm in diameter and 8 mm thick, with 20 sides and a side length of 7.6 mm (Figures 7a,b and S9). This fabrication method allows for tuning mechanical properties of the ring that enables effective microneedle insertion on tissue. Microneedle arrays were fitted on to the 20-sided polygon on the surface of the IVR. Thus, an IVR with 20 microneedle arrays (H = 600 μm) held ∼0.06 mg clotrimazole/ring for liposome and ∼0.1 mg clotrimazole/ring for LBFs. Using the LBF-loaded, 1000 μm microneedle arrays, the highest clotrimazole dose (0.3 mg) could be delivered. These doses are still lower than the currently marketed creams (e.g., Canesten at 10 mg/g), and future studies may explore the incorporation of nanocrystals to further enhance drug payloads within the microneedle tips.^27,55^ However, as shown in this study, microneedles could significantly lower the API dose required to achieve a therapeutic effect (Figure 5c). The proof-of-concept model paves the way for future design optimization and characterization of 3D printed, IVR-based microneedle applicators for treatment of VVC.

**Figure 7:**
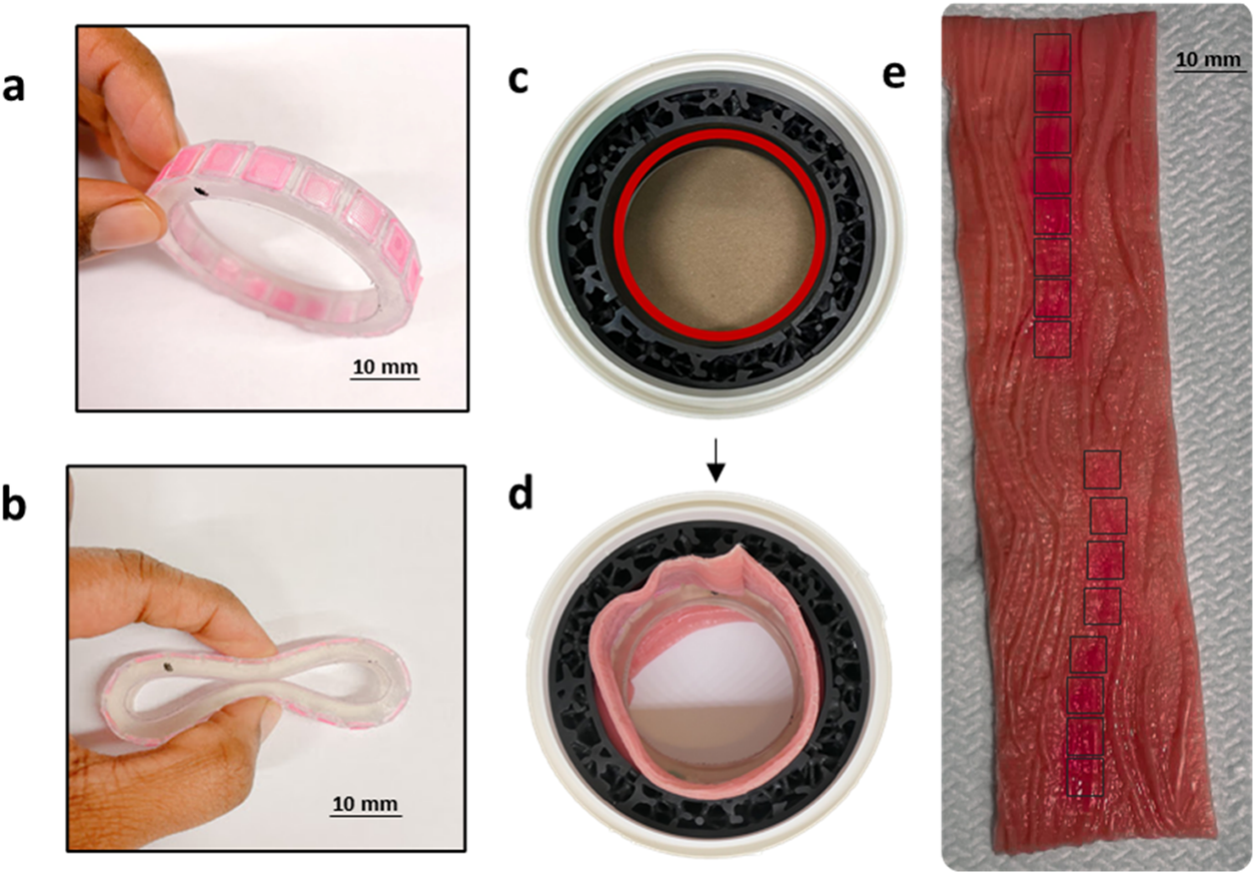
Prototype of a 3D-printed, flexible intravaginal ring (IVR) with antifungal LNC-loaded microneedle arrays (pink) on the surface (a,b). The IVR has a diameter of 56 mm and incorporates 20 microneedle arrays glued to its surface. IVR insertion testing was performed using a 3D-printed ring insertion device (c), where the rigid outer wall (white) had a diameter of 87 mm and the elastic inner lattice (black) had a thickness of 10 mm. A synthetic vaginal tissue lining was placed on the inner surface of the model (d). Upon IVR insertion (e), 16 distinct pink patches corresponding to microneedle arrays can be observed on the tissue surface, indicating successful insertion.

Microneedle insertion was evaluated using a custom 3D-printed ring insertion model (Figures 7c and S10). The model consisted of a rigid outer wall (white) and a flexible inner lattice (black). The inner lining of the model was coated with synthetic vaginal tissue (Figure 7d). For insertion testing, the microneedle-coated IVR prototype containing 16 microneedle arrays and four empty cavities to facilitate handling, was positioned within the tissue-lined model. The IVR was left for 20 min to allow microneedle tip dissolution (Figure 3e). After removal, the synthetic tissue surface displayed 16 distinct pink patches (Figure 7e), corresponding to microneedle insertion from the sulforhodamine B-loaded tips, confirming successful insertion of the arrays covering the IVR. Given that IVRs are intended for self-insertion and removal by the patient, these findings suggest that the device can be removed 1 day postinsertion, by which time microneedle tips have fully dissolved and the nanocarriers have been deposited into vaginal mucosa (Figure 3e). The released nanocarriers enable sustained drug release over an extended period (Figure 2b), potentially reducing the need for frequent reapplication. However, the optimal dose per IVR administration and dosing frequency must be determined in future *in vivo* studies.

Future studies should further characterize the IVR prototype, including mechanical strength testing using a texture analyzer and drug release assessment with the device suspended in simulated vaginal fluid.^56,57^ The *in vitro* model introduced by Teworte et al.,^58^ which simulates systemic drug transport from vaginal ovules, offers a potential framework for biorelevant evaluation. Such studies will further inform the design and refinement of the microneedle-coated IVR and support its detailed assessment in relevant animal models.

## 4 Conclusions

Clotrimazole was formulated into LNCs to address its low water solubility. The hydrodynamic diameter of clotrimazole-loaded liposomes and LBFs were both <500 nm exhibiting good physical storage stability, rendering them suitable for vaginal drug delivery. The antifungal activity of the LNCs against the two most commonly occurring fungal strains *C. albicans* and *C. glabrata* was demonstrated. The clotrimazole-loaded LNCs were successfully incorporated in the tips of rapidly dissolving microneedles of varying needle height with well-defined tip morphology. The microneedles could effectively penetrate and deposit their cargo in bovine vaginal tissue from the dissolving tips. The LNC-loaded microneedles could more effectively inhibit *C. albicans* growth both *in vitro* and *ex vivo* compared to the LNCs alone. Furthermore, the LNC-loaded microneedles reduced *C. albicans* CFU to a greater extent compared to a commercial clotrimazole cream, and at an overall lower drug dose. Finally, an IVR design was presented that can deliver multiple microneedle arrays on its surface in the vaginal tract. In summary, this study illustrates the use of LNC-loaded microneedles, combining the advantages of LNCs to solubilize poorly water-soluble drugs with the flexible design of microneedles to penetrate the mucus barrier and disrupt fungal biofilms. When delivered with an IVR, these microneedles could withstand physiological barriers like the vaginal washout mechanism, while the LNCs would enable prolonged release of clotrimazole at the target site. This could pave the way for more effective treatments of VVC, lowering the therapeutic window and combatting fungal resistance.

## Supplementary Material



## Notes

Detailed composition of clotrimazole-loaded LBF (Table S1); hydrodynamic diameter and drug loading stability over 2 weeks (Tables S2 and S3); microneedle height measurements after dissolution at different time points obtained by SEM (Table S4 and Figure S4); detailed histological scoring provided by the histopathologist (Table S5); cumulative drug release and drug release per unit area over time (Figures S1 and S2); *ex vivo* drug permeation of the LNCs using Franz diffusion cells (Figure S3); *in vitro* inhibition zone diameters against *C*. *albicans* for microneedle patches of three different needle heights (Figure S5); *ex vivo* inhibition of *C*. *albicans* biofilms by Canesten assessed by confocal microscopy (Figure S6); *ex vivo* inhibition of LNCs (Figure S7) and LNC-loaded microneedle patches of three different needle heights (Figure S8); and CAD sketches of the 3D-printed intravaginal ring prototype and ring insertion model (Figures S9 and S10) (PDF)
