# Supplementary material for: Dissolving Microneedles Loaded with Lipid Nanocarriers for Vaginal Delivery of Clotrimazole: In Vitro and Ex Vivo Evaluation
Source: Mol Pharm. 2026 Mar 16;23(4):2611–25. doi: 10.1021/acs.molpharmaceut.5c01721 (PMC13058878; doi:10.1021/acs.molpharmaceut.5c01721)
Supplement: Supplementary file 1 [file mp5c01721_si_001.pdf]

Supporting information

Dissolving Microneedles Loaded with Lipid Nanocarriers for Vaginal Delivery of Clotrimazole: *In vitro* and *Ex vivo* Evaluation

*Paarkavi Udayakumar[1], Nataša Škalko-Basnet[2], Veronica Rondahl[3], Cristhian Fernando Salas Cotaquispe[1], Lisa Myrseth Hemmingsen[2], Georgios A. Sotiriou[4,5], Juan Du[4], and Alexandra Teleki[1]**

[1]Department of Pharmacy, Science for Life Laboratory, Uppsala University, 751 23 Uppsala, Sweden.

[2]Department of Pharmacy, University of Tromsø The Arctic University of Norway, Universitetsveien 57, 9037 Tromsø, Norway.

[3]Department of Animal Biosciences, Section for Anatomy, Physiology, Immunology and Pathology, Swedish University of Agricultural Sciences, Uppsala, Sweden.

[4]Department of Microbiology, Tumor and Cell Biology, Karolinska Institute, 171 77 Stockholm, Sweden.

[5]Department of Chemistry, Stockholm University, 106 91, Stockholm, Sweden.

*Email: alexandra.teleki@scilifelab.uu.se

Table S1. Composition of the clotrimazole-loaded lipid-based formulation (LBF).

**Ingredient**	**% w/w**
Labrafac lipophile	9.1
Labrasol ALF	43.9
Capryol 90	11.0
Propylene glycol	30.0
Benzyl alcohol	1.0
Clotrimazole	5.0
**Total**	100.0

Table S2. Hydrodynamic diameter and polydispersity index (PDI) of as-prepared clotrimazole-loaded lipid-based nanocarriers (LNCs) and after storage for two weeks at room temperature (mean of n=3).

	**Hydrodynamic diameter (nm) ± SD (Week 0)**	**Hydrodynamic diameter (nm) ± SD (Week 2)**	**PDI (Week 0)**	**PDI (Week 2)**
Liposome	170 ± 10	180 ± 13	0.12	0.14
LBF	345 ± 80	350 ± 60	0.28	0.32

Table S3. Clotrimazole load of LNCs as-prepared and after storage for two weeks at room temperature (mean of n=3).

	**Drug load ± SD Week 0**	**Drug load ± SD Week 2**
Liposome (mg/mL)	1.5 ± 0.25	1.48 ± 0.34
LBF (mg/g)	35 ± 2.3	36 ± 1.45

Table S4. Heights of microneedle tips of all heights, at the different timepoints post insertion on excised bovine vaginal tissue (mean of n=3).

**Time**	**H_0 = 600 µm**	**H_0 = 800 µm**	**H_0 = 1000 µm**
0	585 ± 10	790 ± 8	978 ± 13
5 minutes	401 ± 20	460 ± 35	453 ± 33
8 hours	360 ± 42	350 ± 60	332 ± 28
24 hours	280 ± 35	325 ± 26	255 ± 25

Table S5. Histological scoring of explant treatment groups, assessing epithelial damage, inflammation, and presence of fungal hyphae or yeast buds from the epithelial surface to the muscularis. Treatment groups: (a) Day 0 explant, (b) negative control (uninoculated), (c) positive control (fungal-inoculated), (d) liposome-treated, (e) lipid-based formulation (LBF)-treated, and (f–h) blank microneedle patches (H1000, H800, H600).

	Epithelial disruption				Inflammation				Fungal hyphae						Fungal yeast forms					
Treatment group	Attenuated	Eroded	Ulcerated	Single cell death	Intraepitheli al	Lamina propria	Submucosa	Muscularis	Surface	Lamina propria	Submucosa upper third	Submucosa middle third	Submucosa lower third	Muscularis	Surface	Lamina propria	Submucosa upper third	Submucosa middle third	Submucosa lower third	Muscularis
1-1	2	3	0	1	2	2	1	1-2	0	0	0	0	0	0	0	0	0	0	0	0
1-2	2	3	0	1	2	2	1	1-2	0	0	0	0	0	0	0	0	0	0	0	0
1-3	2	3	0	1	2	2	1	1-2	0	0	0	0	0	0	0	0	0	0	0	0
1-4	2	3	0	1	3	3	1	1-2	0	0	0	0	0	0	0	0	0	0	0	0
2-1	3	0	0	3	1	1	0	0	0	0	0	0	0	0	0	0	0	0	0	0
2-2	1	0	0	3	1	1	0	0	0	0	0	0	0	0	0	0	0	0	0	0
2-3	Excluded – extensive areas devitalized, with aggregates of small coccoid bacteria-like infiltrates on surface, in lamina propria and submucosa																			
3-1	4	4	2	3-4	-	1	2	1	2	2	2	2	0	0	2	2-3	2	2	0	0
3-2	4	4	2	3-4	-	1	2	0	2	2	2	0	0	0	3	3	2	0	0	0
3-3	4	4	2	3-4	-	1	2	0	2	2	2	2	0	0	3	3	2	0	0	0
3-4	4	4	2	3-4	-	1	2	1	2	2	2	2	0	0	2	2-3	2	2	0	0
4-1	4	4	2	3-4	-	1	1	0	3	2	0	0	0	0	3	3	0	0	0	0
4-2	4	4	2	3-4	-	1	1	0	3	2	0	0	0	0	3	3	0	0	0	0
4-3	4	4	2	3-4	-	1	1	0	3	2	0	0	0	0	3	3	0	0	0	0
4-4	4	4	2	3-4	-	1	1	0	3	2	0	0	0	0	3	3	0	0	0	0
5-1	4	4	2	3-4	-	2	1	1-2	3	2-3	0	0	0	0	3	2-3	0	0	0	0
5-2	4	4	2	3-4	-	2	2	1	3	3	1-2	1-2	0	0	3	3	1-2	1-2	0	0
5-3	4	4	2	3-4	-	2	2	1	3	3	1-2	1-2	0	0	3	3	1-2	1-2	0	0
5-4	4	4	2	3-4	-	2	2	1	3	3	1-2	1-2	0	0	3	3	1-2	1-2	0	0
5-5	4	4	2	3-4	-	2	2	1	3	3	1-2	1-2	0	0	3	3	1-2	1-2	0	0
5-6	4	4	2	3-4	-	2	2	1	3	3	1-2	1-2	0	0	3	3	1-2	1-2	0	0
6-1	Excluded – aggregates of small coccoid bacteria-like infiltrates on surface, in lamina propria and submucosa																			
6-2	4	4	2	3	-	1	1	1	3	2	1	0	0	0	3	2-3	1-2	0	0	0
7-1	4	4	2	3	-	1	1	1	1	1	1	0	0	0	3	2-3	1-2	0	0	0
7-2	4	4	2	2	-	1	0	0	0	0	0	0	0	0	0	0	0	0	0	0
7-3	4	4	2	2	-	1	0	0	0	0	0	0	0	0	0	0	0	0	0	0
7-4	4	4	2	3	-	1	1	1	1	1	1	0	0	0	3	2-3	1	0	0	0
7-5	4	4	2	2	-	1	0	0	0	0	0	0	0	0	0	0	0	0	0	0
8-1	3	3	1	1	1	0	0	0	0	0	0	0	0	0	0	0	0	0	0	0
8-2	3	3	1	2	1	0	0	0	0	0	0	0	0	0	0	0	0	0	0	0
8-3	3	3	1	2	1	0	0	0	0	0	0	0	0	0	0	0	0	0	0	0
8-4	3	3	1	2	1	0	0	0	0	0	0	0	0	0	0	0	0	0	0	0
8-5	3	3	1	2	1	0	0	0	0	0	0	0	0	0	0	0	0	0	0	0
8-6	3	3	1	2	1	0	0	0	0	0	0	0	0	0	0	0	0	0	0	0

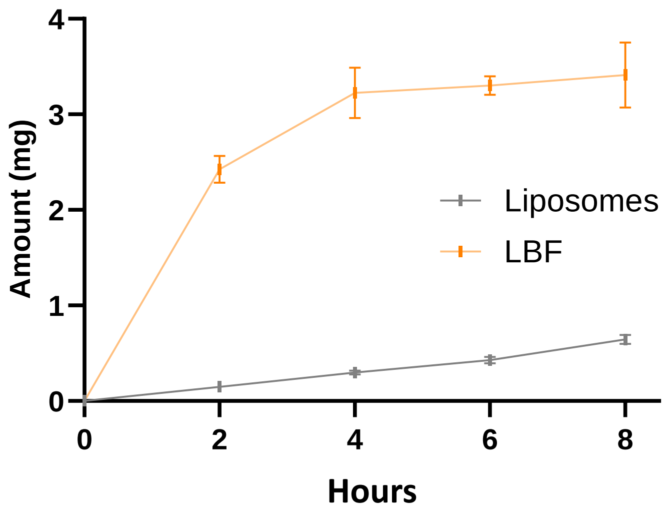

Figure S1. *In vitro* cumulative drug release of the LNCs in amounts over time (mean of n=3).

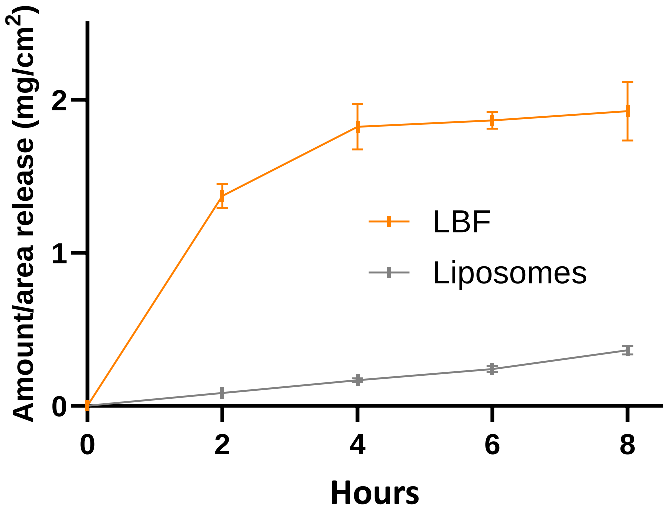

Figure S2. *In vitro* cumulative drug release of the LNCs per area over time (mean of n=3).

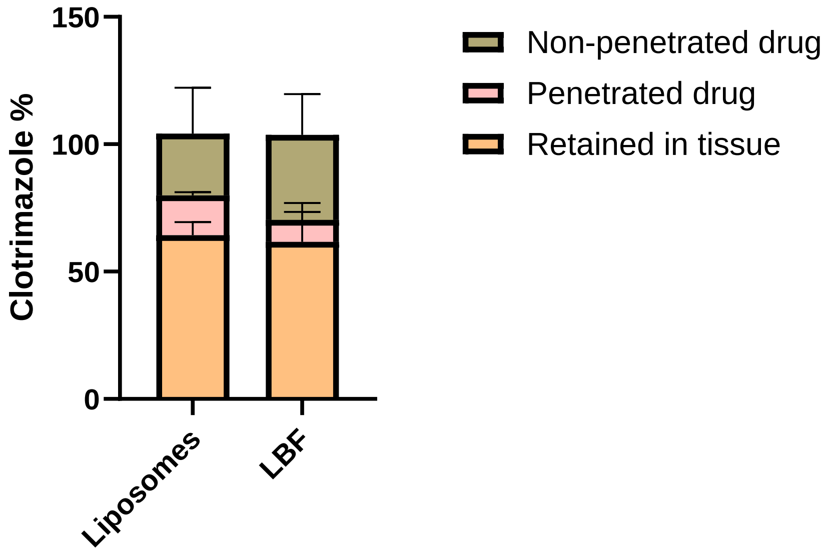

Figure S3. *Ex vivo* drug permeation of the LNCs (Mean of n=3).

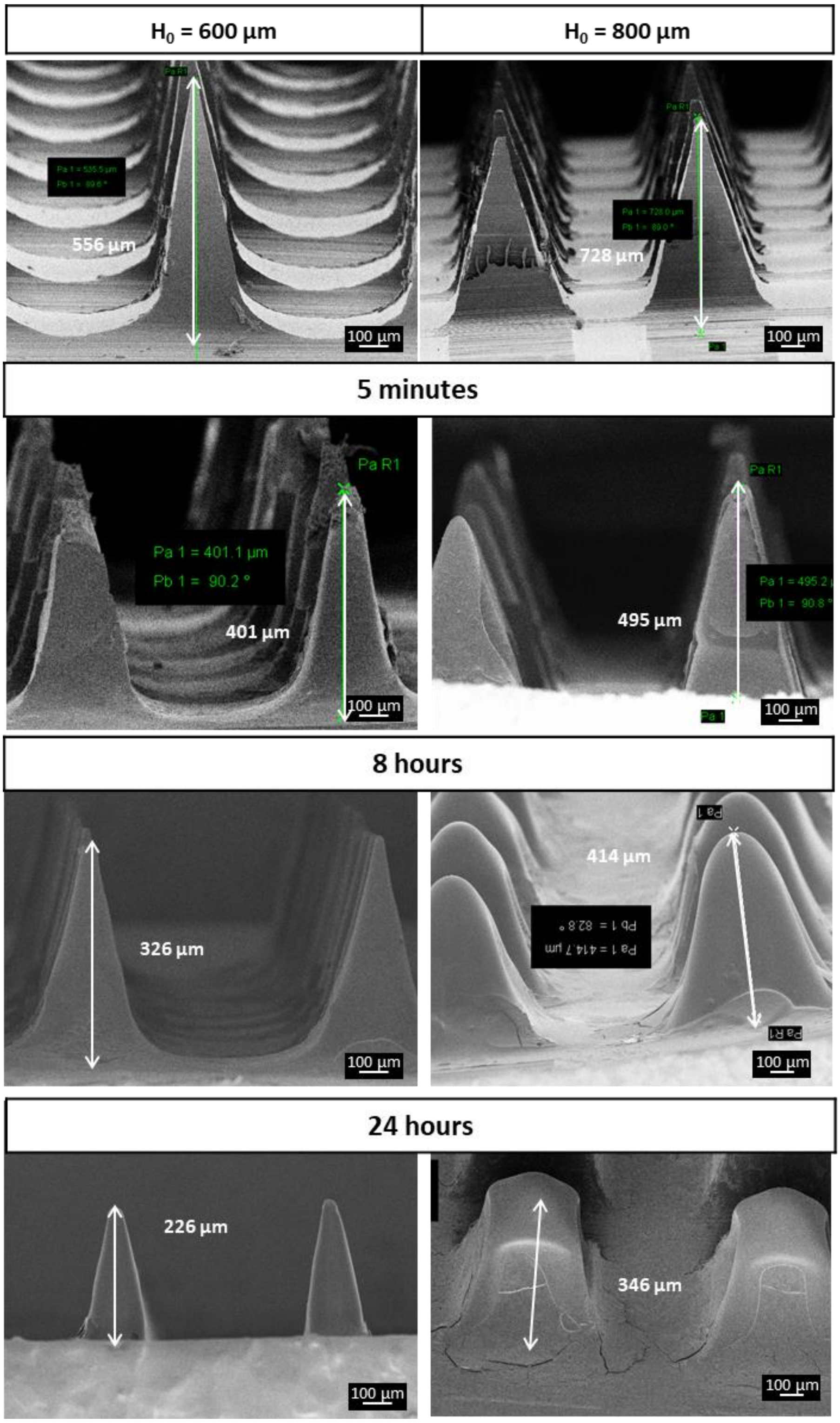

Figure S4. SEM images of microneedle tips of height 600 μm and 800 μm at the different timepoints post insertion on excised bovine vaginal tissue.

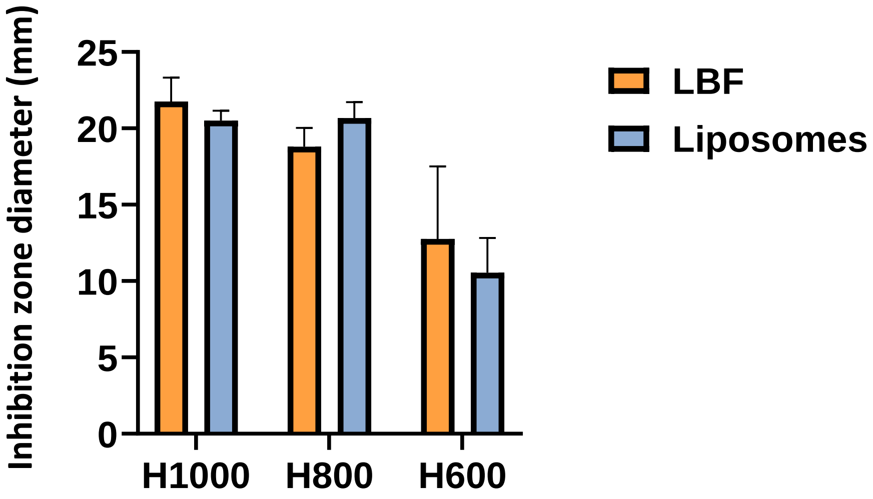

Figure S5. Inhibition zone diameters (in mm) against *C. albicans* of LNC-loaded microneedle arrays for three tip heights: (H) 600, 800 and 1000 µm. Standard deviations are indicated, n=3.

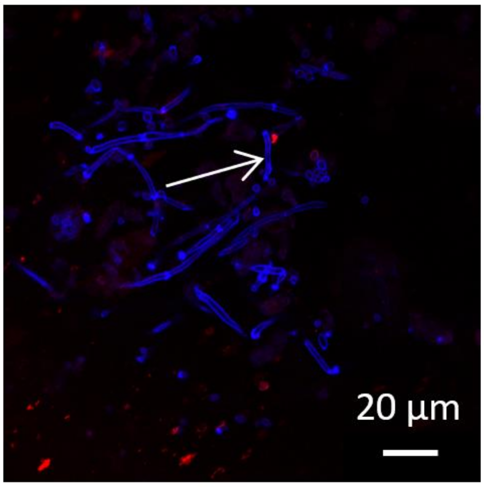

Figure S6. Confocal microscopy of the fungal biofilm (in blue) with candida buds and hyphae (indicated by a white arrow) on an explant infected with *C. albicans* after treatment with Canesten cream. The extra-cellular matrix is stained in red.

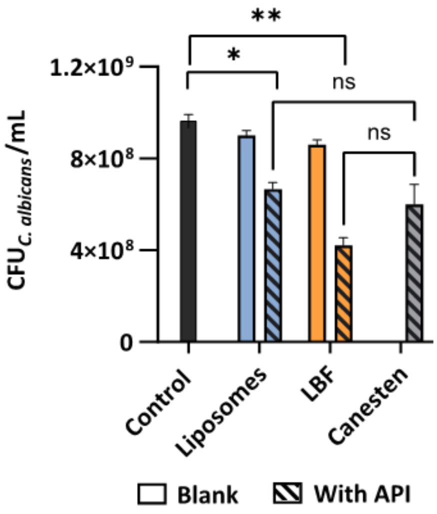

Figure S7. CFU$_{C.\ albicans}$ after treatment with blank (solid fill) and clotrimazole-loaded (dashed lines) LNCs for 24 hours and the untreated fungal explant as control. p < 0.05 (*), p <0.005 (**), p<0.0005 (***) and ns > 0.05.

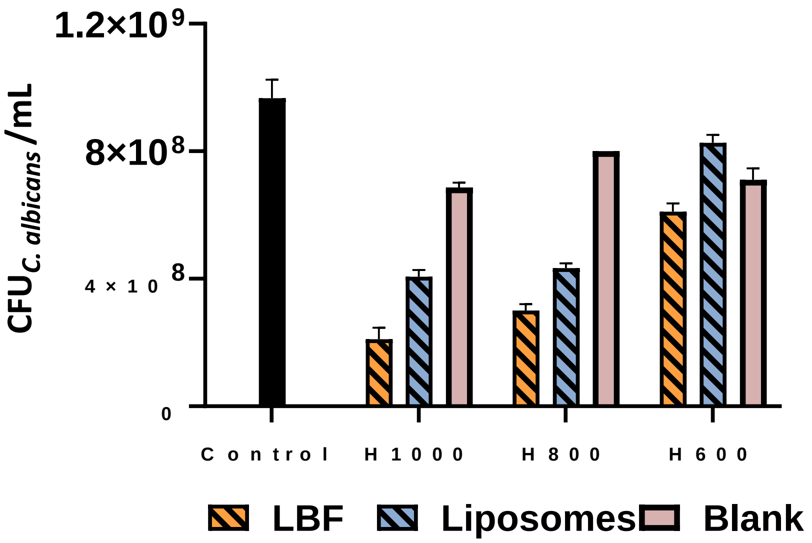

Figure S8. *Ex vivo* antifungal activity (measured in CFU/mL) against *C. albicans* on the explant model of blank and LNC-loaded microneedle arrays at three tip heights (H) 600, 800 and 1000 µm, control being the untreated explant.

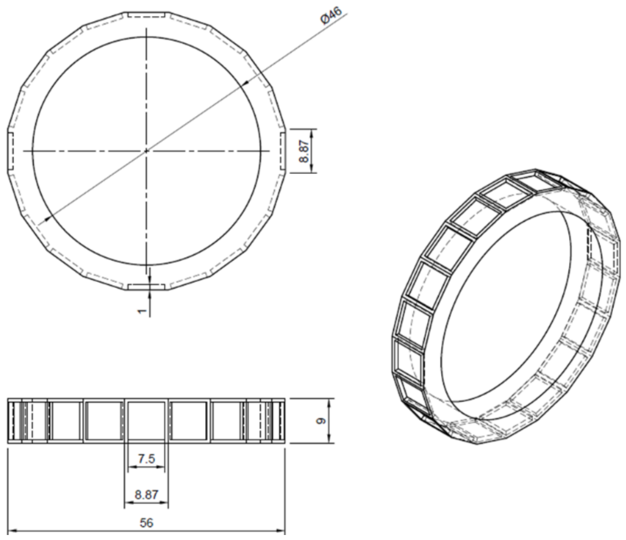

Figure S9. Dimensions of the 3D printed IVR prototype.

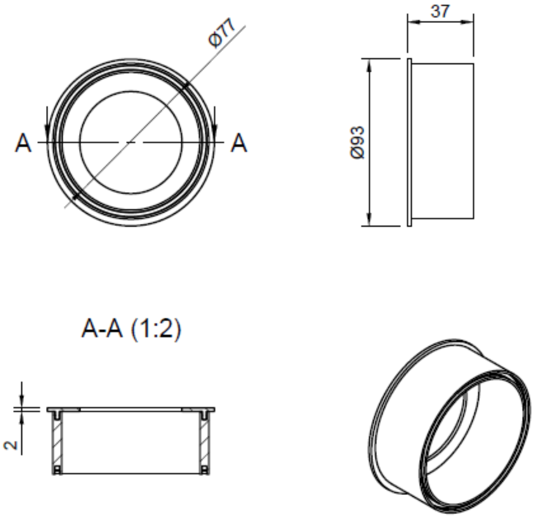

Figure S10. Dimensions of the 3D printed ring insertion model.
